# Integrated Machine Learning and Statistical Interpretation of Malachite Green Removal by SDBS-Modified Ostrich Bone Waste: A Comparative Random Forest, Bayesian Regression, and LMG Analysis

**DOI:** 10.3390/molecules31193484

**Published:** 2026-09-29

**Authors:** Mehdi Bahrami, Mohammad Javad Amiri, Zahra Rahsepar Chargi, Barbara Lednicka, Mohammad Gheibi

**Affiliations:** 1 Department of Water Engineering, Faculty of Agriculture, Fasa University, Fasa 74616-86131, Iranrchargi02m@fasau.ac.ir (Z.R.C.); 2 Research Institute of Water Resources Management in Arid Region, Fasa University, Fasa 74616-86131, Iran; 3 Department of Physics, Faculty of Marine Engineering, Gdynia Maritime University, 81-225 Gdynia, Poland; 4 Institute for Nanomaterials, Advanced Technologies and Innovation, Technical University of Liberec, Studentská 1402/2, 461 17 Liberec, Czech Republic

**Keywords:** malachite green, ostrich bone waste, SDBS modification, adsorption, Bayesian regression, Random Forest, Lindeman–Merenda–Gold analysis, machine learning, wastewater treatment

## Abstract

The development of sustainable adsorbents and reliable predictive tools for dye-contaminated wastewater treatment remains an important challenge in environmental engineering. In this study, the adsorption of malachite green (MG) dye onto sodium dodecyl benzene sulfonate (SDBS)-modified ostrich bone waste was investigated using an integrated modeling framework combining Lindeman–Merenda–Gold (LMG) analysis, Bayesian Regression (BR), and Random Forest (RF) modeling. 52 experimental conditions (each measured in triplicate) were analyzed to evaluate the influence of pH, contact time, initial dye concentration, temperature, adsorbent dosage, and SDBS dosage on dye removal efficiency. The three modeling approaches consistently identified pH and contact time as the two most influential operational variables, whereas the relative importance of initial dye concentration and adsorbent dosage differed among the methods. BR quantified the direction and uncertainty of predictor effects, yielding posterior coefficients of 0.650 (95% CrI: 0.365–0.935) for pH, 0.467 (95% CrI: 0.263–0.672) for contact time, and −0.517 (95% CrI: −0.759 to −0.272) for initial dye concentration. Under internal validation, RF yielded an OOB R^2^ of 0.822, an RMSE of 11.79 pp, and an MAE of 7.85 pp, whereas BR evaluated by LOOCV yielded an R^2^ = 0.417, an RMSE = 21.31 pp, and an MAE = 16.42 pp. Because different validation protocols were used, these internal estimates are not directly comparable. More stringent group-aware validation resulted in under leave-one-series-out validation; neither model achieved positive skill (pooled R^2^ = −0.382 for RF; BR R^2^ was strongly negative, driven mainly by extrapolation when the initial-concentration series was held out), while across the six single-series rotations, R^2^ was negative in five for both models (RF positive only when the C_0_ series was held out; BR best when the pH series was held out, R^2^ = 0.410). These results demonstrate that the models capture relationships within the investigated experimental domain but have limited generalization to experimental series or operating regions not represented in model development. The results confirm the potential of SDBS-modified ostrich bone waste (OBW) as an effective and sustainable adsorbent for malachite green removal and demonstrate that the proposed integrated modeling framework therefore enabled complementary assessment of variable importance, probabilistic predictor effects, and predictive behavior within and across the investigated experimental domain.

## 1. Introduction

Synthetic dyes are among the most persistent pollutants released into aquatic environments due to their extensive use in textile, printing, aquaculture, pharmaceutical, and food-processing industries. Among these contaminants, malachite green (MG) has received considerable attention because of its high stability, resistance to biodegradation, and potential toxic, mutagenic, and carcinogenic effects on living organisms. Although MG is not a bulk constituent of municipal wastewater, it remains a contaminant of significant environmental and public health concern because of its continued use in aquaculture, textile dyeing, leather processing, and paper printing, combined with its high environmental persistence and documented toxic, mutagenic, and carcinogenic effects. The discharge of MG-containing effluents, even at relatively low concentrations, can adversely affect aquatic ecosystems and human health through food-chain accumulation and drinking-water contamination. Consequently, MG is representative of a broader class of recalcitrant cationic dyes whose removal remains an important challenge in industrial and agricultural wastewater treatment [1,2,3]. Beyond its direct environmental relevance, MG is widely employed as a model cationic dye in adsorption research because its well-defined molecular structure, pH-dependent speciation, and strong electrostatic interactions with anionic surfaces make it an ideal probe for elucidating surface-charge–controlled adsorption mechanisms [1,3,4]. Insights gained from MG adsorption are therefore transferable to other cationic dyes and organic micropollutants that share similar electrostatic and aromatic characteristics [4,5].

Adsorption has emerged as one of the most effective and economically attractive methods for dye removal owing to its operational simplicity, high removal efficiency, and adaptability to various wastewater compositions. In recent years, increasing attention has been directed toward the utilization of low-cost bio-based adsorbents derived from agricultural and animal wastes. Such materials not only reduce treatment costs but also contribute to waste valorization and circular-economy strategies. Animal bone waste, in particular, has attracted interest because of its high mineral content, porous structure, and natural affinity toward various pollutants [1,2,3].

Surface modification represents an effective strategy for improving the adsorption performance of waste-derived adsorbents. Among various modifiers, anionic surfactants such as sodium dodecyl benzene sulfonate (SDBS) can alter the surface chemistry, increase the availability of active adsorption sites, and enhance electrostatic interactions with cationic dyes. Previous studies have demonstrated that surfactant modification can substantially improve the adsorption behavior of bio-based materials toward dye molecules, including MG and related contaminants [4,5].

Alongside experimental investigations, data-driven modeling approaches have become increasingly important for understanding adsorption systems and predicting process performance. Conventional statistical models often assume linear relationships between process variables and adsorption responses, whereas adsorption systems frequently involve nonlinear interactions and complex dependencies among operational factors. Machine-learning techniques have therefore gained significant attention for modeling adsorption processes and identifying the most influential operating variables [6,7,8].

Random Forest (RF) is one of the most powerful ensemble-learning algorithms for capturing nonlinear relationships and variable interactions while maintaining high predictive accuracy [6,9]. Bayesian regression provides a probabilistic framework that incorporates uncertainty quantification and allows robust inference regarding the contribution of individual variables [10]. In contrast, LMG method offers a rigorous variance-decomposition approach for evaluating the relative importance of predictors in multivariate systems [11,12]. The simultaneous application of these complementary methods enables both accurate prediction and interpretable assessment of adsorption mechanisms.

However, despite the individual strengths of these methods, their combined application within a single analytical workflow remains largely unexplored in adsorption research. Specifically, three critical shortcomings persist in the existing literature. First, most adsorption modeling studies rely on a single modeling approach—either a linear regression model, a machine-learning algorithm, or a variance-decomposition method—and therefore cannot simultaneously capture nonlinear interactions, quantify parameter uncertainty, and rigorously rank variable importance [6,7,8]. Second, machine-learning models such as Random Forest are often criticized for their lack of interpretability, while conventional statistical models such as Bayesian regression may fail to capture complex nonlinear relationships; this trade-off between predictive accuracy and mechanistic insight has not been systematically addressed in adsorption studies [9,10]. Third, few studies have integrated variance-decomposition (LMG), probabilistic inference (BR), and nonlinear machine learning (RF) into a unified framework for adsorption-process analysis, despite the clear complementarity of these approaches [11,12]. These shortcomings directly motivate the present study.

Although machine-learning techniques have been widely employed for modeling adsorption processes, studies integrating predictive, probabilistic, and variance-based interpretation approaches within a single framework remain limited. Moreover, limited information is available regarding the adsorption of MG using SDBS-bone waste and the relative importance of the operational parameters governing the process.

The shortcomings identified above—the lack of a unified modeling framework, the trade-off between predictive accuracy and interpretability, and the absence of uncertainty quantification in adsorption modeling—define the specific research problem addressed in this study. To directly address these gaps, the objective of the present study was to investigate the adsorption behavior of MG using SDBS-modified OBW and to establish a comprehensive modeling framework based on Random Forest, Bayesian regression, and LMG analysis. The specific aims were (i) to evaluate the predictive capability of different modeling approaches, (ii) to identify the most influential operational variables affecting dye removal efficiency, (iii) to quantify model uncertainty and variable importance, and (iv) to provide a robust and interpretable framework for adsorption-process analysis that can support the development of sustainable wastewater treatment technologies. By integrating these three complementary methods, the present study directly addresses each of the identified shortcomings: LMG provides rigorous variable-importance ranking, BR supplies probabilistic interpretation and uncertainty quantification, and RF provides flexible nonlinear modelling, whose predictive skill is tested here under both row-wise and group-aware validation.

### Research Hypotheses and Core Contribution

The central hypothesis of this study is that an integrated modeling framework combining LMG, Bayesian Regression, and Random Forest provides complementary and mutually reinforcing advantages that cannot be achieved by any single modeling approach. Specifically, we hypothesize that:

(i) LMG analysis can rigorously quantify the relative importance of operational variables by averaging variance decomposition across all predictor orderings, thereby overcoming the order-dependency limitation of conventional regression;
(ii) BR can provide probabilistic interpretation of predictor effects through posterior distributions and credible intervals, enabling uncertainty quantification that is absent in deterministic models;
(iii) RF can capture nonlinear and interaction effects and may achieve higher within-design predictive accuracy than a linear model; whether this extends across experimental series is evaluated with group-aware validation;
(iv) The integration of these three approaches enables simultaneous identification of dominant variables, probabilistic interpretation of predictor effects, and assessment of predictive behaviour within and across the experimental domain—thereby providing a more robust and interpretable framework than any single method alone.

The SDBS-OBW system is employed as a representative hydroxyapatite-rich bio-waste adsorbent to demonstrate the integrated framework. The material is not claimed as the primary novel contribution; rather, it serves as a well-characterized case study on which the framework’s advantages are evaluated and validated. The principal significance of this study lies not solely in the removal of MG by a specific adsorbent, but in the development and evaluation of a framework that combines LMG, BR, and RF within a single analytical workflow. This framework addresses a persistent gap in adsorption research: the lack of a unified approach that simultaneously provides (i) rigorous variable-importance ranking, (ii) probabilistic quantification of predictor effects and uncertainty, and (iii) evaluation of predictive performance both within and beyond the experimental domain. Although demonstrated here for MG adsorption onto SDBS-OBW, the framework is transferable to other dye–adsorbent systems, other classes of micropollutants, and other wastewater treatment processes where multiple interacting variables govern performance.

## 2. Materials and Methods

### 2.1. Preparation of SDBS-Modified Ostrich Bone Waste

The OBW utilized in this study was derived from bone waste procured from a local butcher supplier in Fasa, Fars Province, Iran. To eliminate superficial organic residues, fat, and blood clots, the raw bones were thoroughly rinsed with deionized water and subsequently dried. The cleaned bones were then crushed into small fragments and subjected to a defatting pretreatment by immersion in a 1:1 (*v*/*v*) ethanol–water solution under continuous stirring for 2 h at room temperature, followed by repeated rinsing with deionized water until a neutral pH was reached. The pretreated bone fragments were dried in a conventional oven at 105 °C for 24 h, then ground and sieved through a 100-mesh (150 µm) sieve to obtain a homogeneous particle size distribution prior to SDBS modification.

For the synthesis of SDBS-OBW, 5 g of the processed bone waste was accurately weighed and dispersed in 100 mL of distilled water within a 250 mL beaker, corresponding to a solid–liquid ratio of 1:20 (*w*/*v*). The suspension was continuously stirred using a magnetic stirrer at 400 rpm. Varying quantities of SDBS (molecular weight = 348.48 g mol^−1^) were introduced to the mixture to achieve surfactant loadings ranging from 0.5 to 1.5 meq g^−1^. The SDBS loading was calculated from the mass of SDBS introduced during synthesis using the molecular weight of SDBS (348.48 g mol^−1^) and the fact that each SDBS molecule carries one negative charge, corresponding to 1 equivalent per mole of SDBS. The loading was expressed on a total-mass basis (bone + immobilized SDBS), assuming complete immobilization of the introduced surfactant. The resulting slurry was subjected to mechanical agitation on a heated plate set at 70 °C for a duration of 1 h to facilitate the adsorption and intercalation of the anionic surfactant onto the bio-apatite surface. Post-modification, the solid phase was isolated via centrifugation at 4000 rpm for 10 min. The recovered pellet was then washed twice with a 50% (*v*/*v*) aqueous ethanol solution to remove any excess, unbound SDBS molecules. The purified material was subsequently dried in a conventional oven at 60 °C for 12 h (overnight). Finally, the dried product was ground into a fine powder using an agate mortar and pestle and sieved through a 100-mesh (150 µm) sieve to obtain a homogeneous particle size distribution. The resulting SDBS-OBW was stored in an airtight container for subsequent adsorption experiments.

### 2.2. Batch Adsorption Experiments

The experimental dataset comprised 52 distinct batch adsorption conditions representing different combinations of six operational variables affecting MG removal by SDBS-OBW. Each experimental condition was measured in triplicate (*n* = 3), and the mean removal efficiency was used as the response value for subsequent statistical and machine-learning analyses. Batch adsorption experiments were conducted in 250 mL Erlenmeyer flasks containing 100 mL of MG solution of the desired concentration. The flasks were placed in a temperature-controlled shaker at a constant agitation speed of 150 rpm. The investigated variables were solution pH (2–11), contact time (0–150 min), initial MG concentration (C_0_, 5–150 mg L^−1^), SDBS dosage (0–1.5 meq g^−1^), adsorbent dosage (0–1.0 g L^−1^), and temperature (25–55 °C). The solution pH was adjusted using 0.1 M HCl or 0.1 M NaOH and monitored with a calibrated pH meter (Metrohm 827 pH Lab; Metrohm, Herisau, Switzerland). Following each batch experiment, the residual MG concentration in the aqueous phase was determined using UV–visible spectrophotometric analysis (Shimadzu UV-1800; Shimadzu, Kyoto, Japan) at a wavelength of 617 nm. The removal efficiency (*R*) was calculated from the initial and equilibrium MG concentrations:

(1) $$R=\left(\frac{C_{0}-C_{e}}{C_{0}}\right)\times 100$$

In this context, *C*_0_ and *C_e_* refer to the MG initial concentration and the concentration at equilibrium (measured in mg/L).

The 52 experiments were arranged as six one-variable-at-a-time (OVAT) series, each measured as a separate sequential experiment on the same adsorbent batch and solution system: (i) pH 2–11 at fixed contact time (120 min), *C*_0_ (20 mg L^−1^), SDBS dosage (0.5 meq g^−1^), adsorbent dosage (0.1 g L^−1^) and temperature (25 °C; 10 runs); (ii) contact time 0–150 min at pH 9 under the same background conditions (11 runs); (iii) *C*_0_ 5–150 mg L^−1^ at pH 9, contact time 120 min, SDBS dosage 0.5 meq g^−1^, adsorbent dosage 1 g L^−1^ and 25 °C (9 runs); (iv) SDBS dosage 0–1.5 meq g^−1^ at pH 2, contact time 120 min, *C*_0_ 50 mg L^−1^, adsorbent dosage 1 g L^−1^ and 55 °C (7 runs); (v) adsorbent dosage 0–1.0 g L^−1^ at pH 9, contact time 120 min, *C*_0_ 20 mg L^−1^, SDBS dosage 0.5 meq g^−1^ and 25 °C (11 runs); and (vi) temperature 25–55 °C at pH 9, contact time 120 min, *C*_0_ 50 mg L^−1^, SDBS dosage 0.5 meq g^−1^ and adsorbent dosage 1 g L^−1^ (4 runs). Because consecutive runs within a series share the same experimental system and are therefore serially correlated, the experimental series- rather than the individual rows- were treated as the independent blocks in all group-aware validation analyses.

The resulting experimental matrix was subsequently used as the common dataset for LMG analysis, Bayesian regression, and Random Forest modeling, enabling direct comparison of variable importance, parameter effects, and predictive performance among the three approaches. Prior to model development, the dataset was examined for consistency and completeness. The available data were then used to construct predictive and interpretable models aimed at identifying the key factors governing the adsorption process. Because each factor was varied in only one series while the others were held at series-specific values (e.g., adsorbent dose 0.1 or 1.0 g L^−1^; the SDBS series run at pH 2 and 55 °C), factor effects, importance rankings and cross-series predictions are partly confounded with series identity.

### 2.3. Modeling Framework

**Figure 1:**
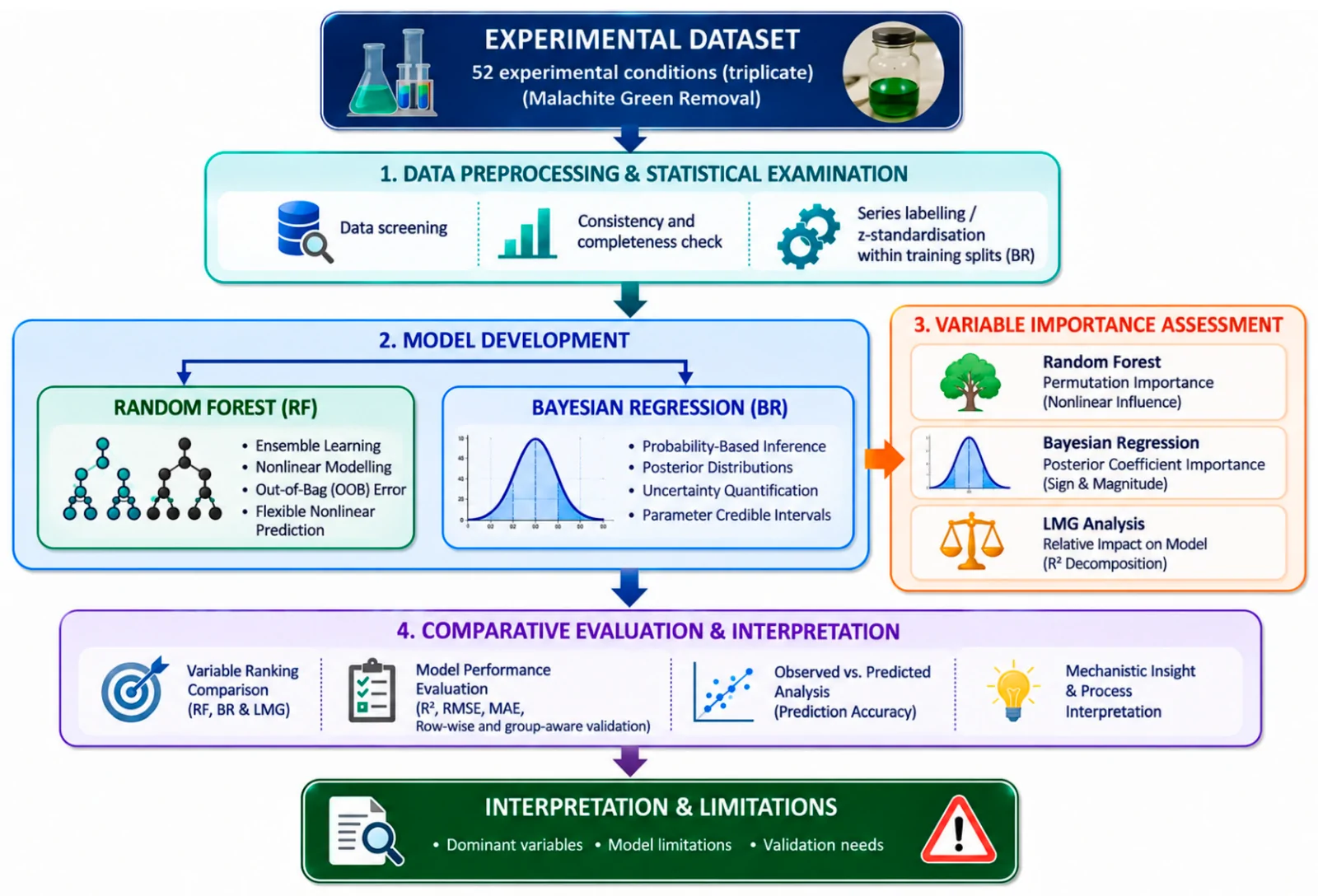
Integrated modeling framework employed for the analysis of malachite green adsorption onto SDBS-modified ostrich bone waste.

A hybrid modeling framework combining Random Forest (RF), Bayesian Regression (BR), and Lindeman–Merenda–Gold (LMG) analysis was employed. The selected methods provide complementary information regarding prediction accuracy, uncertainty quantification, and variable importance. Figure 1 illustrates the overall workflow adopted in this study.

The workflow consisted of four major steps:

1. Data preprocessing and statistical examination.
2. Development of RF and BR models.
3. Variable-importance assessment using RF, BR, and LMG methods.
4. Comparative evaluation of model performance and interpretation of the adsorption process.

### 2.4. Random Forest Modeling

Random Forest is an ensemble machine-learning algorithm based on the aggregation of multiple decision trees. The method constructs many regression trees via bootstrap sampling and random feature selection, and the final prediction is obtained by averaging their outputs. Owing to its ability to capture nonlinear relationships and complex interactions among variables, RF has been widely applied in environmental and adsorption studies [6,13].

For a dataset containing p predictor variables, each tree was constructed using a random subset of predictors selected at each node. The final prediction was calculated as [9,14]:

(2) $$\hat{Y}=\frac{1}{N_{t}}\sum_{i=1}^{N_{t}}T_{i}\left(x\right)$$

where $\hat{Y}$ is the predicted response, N_t_ is the number of trees, and T_i_(x) denotes the prediction of the *i*th tree.

Out-of-bag (OOB) observations were used to estimate prediction error and assess model generalization. Variable importance was determined using permutation-based importance analysis, where the increase in prediction error caused by random permutation of each predictor was quantified.

All RF computations were performed with n_estimators = 500 regression trees, max_features = 2 candidate predictors evaluated at each split (p/3; the scikit-learn default would use all predictors), bootstrap = True, oob_score = True, and a fixed random seed (random_state = 42) to guarantee reproducibility. The adequacy of the selected ensemble size was verified by monitoring the out-of-bag error as a function of the number of trees.

### 2.5. Bayesian Regression Analysis

Bayesian regression was employed to quantify parameter uncertainty and evaluate the probabilistic influence of operational variables on malachite green removal efficiency. Unlike conventional regression approaches, which treat model parameters as fixed quantities, Bayesian inference considers model parameters as probability distributions and updates prior knowledge using observed data [10,15]. The Bayesian linear regression model can be expressed as [16]:

(3) Y=Xβ+ε

where Y is the response vector, X is the predictor matrix, β represents regression coefficients, and ε is the random error term.

Assuming normally distributed residuals, the likelihood function is defined as:

(4) $$Y|X,\beta ,\sigma^{2}∼N(X\beta ,\sigma^{2}I)$$

where $\sigma^{2}$ is the residual variance and *I* is the identity matrix.

Parameter estimation was performed using Bayes’ theorem, which combines prior information with the observed data to obtain posterior distributions [17]:

(5) $$P(\beta |Y)=\frac{P(Y|\beta )P(\beta )}{P(Y)}$$

where P(β\Y) denotes the posterior distribution of the regression coefficients, P(Y\β) is the likelihood function, P(β) represents the prior distribution, and P(Y) is the marginal likelihood.

Prior distributions were assigned on the z-standardised scale (all predictors and the response centred and scaled to zero mean and unit variance within every training split) as β_j_|σ^2^ ~ N(0, 10^2^) for the intercept and the six regression coefficients, and σ^2^ ~ Inverse-Gamma(a_0_ = 0.001, b_0_ = 0.001) for the residual variance. Posterior inference combined these weakly informative priors with the likelihood (Equation (4)) by means of Bayes’ theorem (Equation (5)) and was implemented through Gibbs sampling with four independent chains, each run for 25,000 iterations; the first 5000 iterations of each chain were discarded as warm-up (burn-in), and the remainder was thinned by a factor of two, yielding 40,000 retained posterior draws (4 chains × 10,000). Posterior means, medians, standard deviations and 95% credible intervals for all model coefficients were computed from the retained draws. The complete model specification is summarised in Table S1.

Convergence of the chains was assessed using the split potential scale reduction factor (split $\hat{R}$), for which values below 1.01 were considered indicative of convergence a priori, complemented by the bulk effective sample size (ESS) and visual inspection of trace plots of every parameter. Posterior-predictive checks were performed by generating 2000 replicated data sets from the posterior predictive distribution and comparing the discrepancy statistics of the replicated and observed data (standard deviation, minimum and maximum); in addition, 95% posterior-predictive intervals (PPIs) were constructed to quantify the coverage of the observations.

For comparative interpretation of predictor contributions, variable importance was subsequently derived from the posterior coefficient estimates and compared with the importance measures obtained from the Random Forest and LMG analyses.

### 2.6. Lindeman–Merenda–Gold (LMG) Analysis

The LMG is a variance-decomposition approach used to quantify the relative contribution of individual predictors in multiple regression models. Unlike conventional regression coefficients, the LMG method accounts for the order-dependency problem by averaging the incremental coefficient of determination (R^2^) across all possible predictor orderings [12,18]. For a predictor X_i_, the LMG importance measure is defined as:

(6) $$LMG_{i}=\frac{1}{p!}\sum_{r}\Delta R_{i}^{2}\left(r\right)$$

where p is the number of predictors and $\Delta R_{i}^{2}\left(r\right)$ represents the increase in explained variance attributed to predictor *i* under ordering *r*.

### 2.7. Model Evaluation

The predictive performance of the developed models was evaluated using several statistical indicators commonly employed in adsorption modeling and machine-learning studies. The coefficient of determination (R^2^), root mean square error (RMSE), and mean absolute error (MAE) were used to assess the agreement between experimental and predicted removal efficiencies.

The coefficient of determination measures the proportion of variance in the response variable explained by the model. The root mean square error, which quantifies the average magnitude of prediction errors while assigning greater weight to larger deviations, was calculated as:

(7) $$RMSE=\sqrt{\frac{1}{n}\sum_{i=1}^{n}{\left(Y_{i}-{\hat{Y}}_{i}\right)}^{2}}$$

The mean absolute error, representing the average absolute difference between observed and predicted values, was computed using:

(8) $$MAE=\frac{1}{n}\sum_{i=1}^{n}\left|Y_{i}-{\hat{Y}}_{i}\right|$$

where *n* is the total number of observations.

All performance metrics were computed with consistent formulas- R^2^ = 1 − SSres/SStot, and RMSE and MAE, expressed in percentage points (pp)- on a single, explicitly labelled set of predictions per reported value. Four prediction sources are distinguished throughout the manuscript: (i) in-bag (training) predictions, which characterise model fit but not predictive performance; (ii) out-of-bag (OOB) predictions, in which each observation of the RF model is predicted exclusively by the trees whose bootstrap sample excluded it; (iii) cross-validation predictions; and (iv) independent test predictions.

Cross-validation was implemented in two complementary ways: a naive row-wise scheme, comprising standard 10-fold CV (repeated 50 times with randomised folds), leave-one-out CV and a random row-wise 75/25 hold-out; and a group-aware scheme, in which the six experimental series were treated as indivisible blocks, comprising leave-one-series-out CV (each series held out once), repeated group-aware 3-fold CV with random series-to-fold assignment (300 repeats), and an independent test set formed by one entire series. The group-aware schemes guarantee that serially correlated runs of the same experimental series never appear simultaneously in the training and evaluation sets. Hyperparameters of both models were kept constant across all schemes (n_estimators = 500, max_features = 2, and random_state = 42 for RF; the priors and MCMC settings for BR), and all metrics were reported without clipping of negative R^2^ values.

In addition to predictive accuracy, model interpretability was assessed through variable-importance analysis. The relative contributions of operational variables were determined using permutation-based importance in the Random Forest model, posterior coefficient analysis in Bayesian regression, and variance decomposition through the LMG approach. The resulting importance rankings were subsequently compared to identify the dominant factors governing malachite green removal efficiency.

### 2.8. Statistical Analysis and Software

All statistical analyses and model development procedures were conducted in Python 3.13. Random Forest models were implemented with scikit-learn 1.6.1 (RandomForestRegressor), whereas Bayesian regression (Gibbs sampler) and LMG analysis were performed using custom Python scripts (NumPy 2.3.5, SciPy 1.17.1). Figures were produced with Matplotlib 3.10.9. Graphical outputs, variable-importance analyses, diagnostic plots, and performance statistics were generated directly from the developed computational framework.

## 3. Results and Discussion

### 3.1. Characterization of SDBS-Modified Ostrich Bone Waste

**Figure 2:**
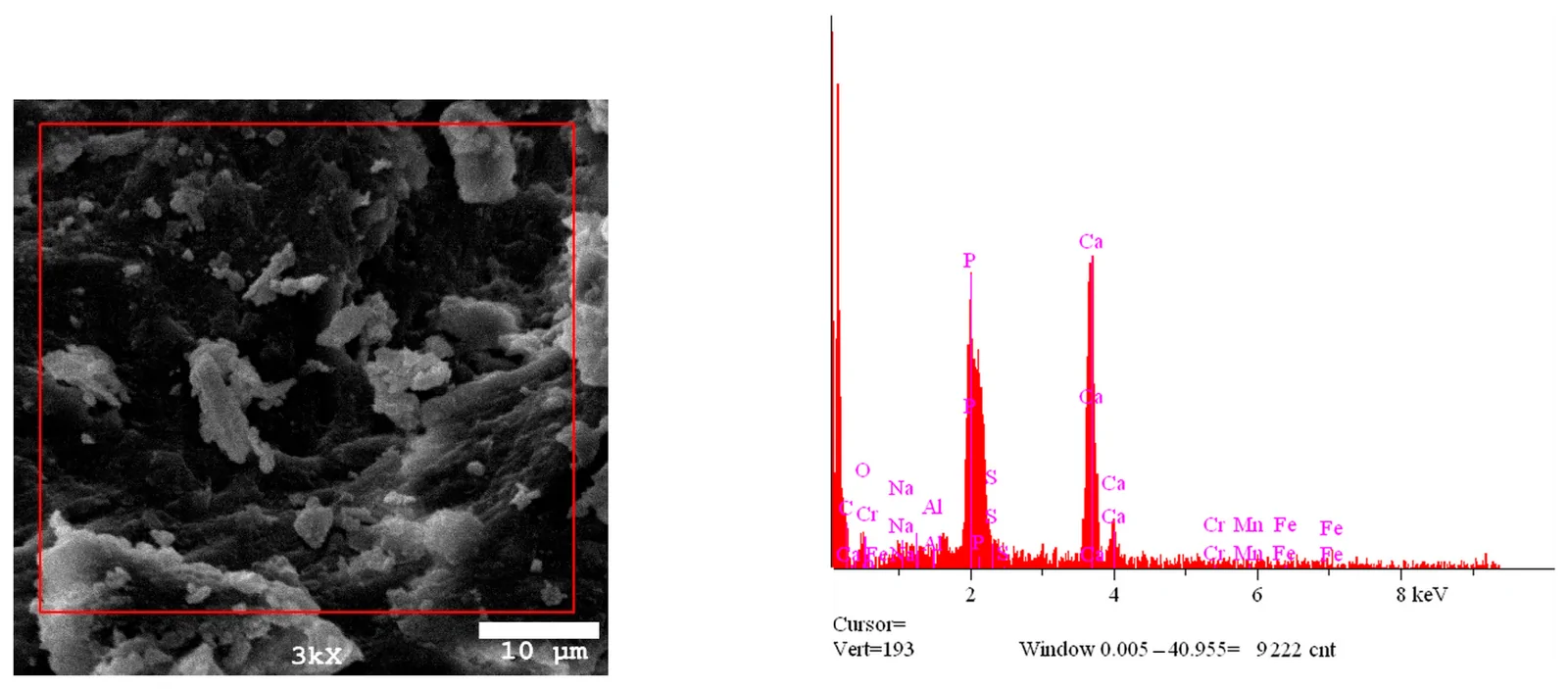
SEM–EDS characterization of SDBS-OBW, showing its surface morphology and elemental composition.

The sodium dodecyl benzene sulfonate (SDBS)-modified ostrich bone waste (SDBS-OBW) was characterized using scanning electron microscopy coupled with energy-dispersive X-ray spectroscopy (SEM–EDS), Fourier-transform infrared spectroscopy (FT-IR), X-ray diffraction (XRD), and thermogravimetric analysis (TGA). SEM–EDS revealed a heterogeneous, folded, and porous surface, with Ca (25.95 wt.%) and P (18.86 wt.%) as the dominant inorganic elements. The atomic Ca/P ratio of 1.06 is below the stoichiometric value for hydroxyapatite (1.67), and EDS is only semi-quantitative. The detection of S (2.83 wt.%) and Na (2.51 wt.%) is compatible with an SDBS-derived sulfonate component (Figure 2). Sulfur is not a structural constituent of hydroxyapatite, so its detection (2.83 wt.%) is compatible with, but does not prove, the presence of SDBS, because no EDS of raw OBW was available for comparison. The results of the EDS analysis are presented in Table S2.

**Figure 3:**
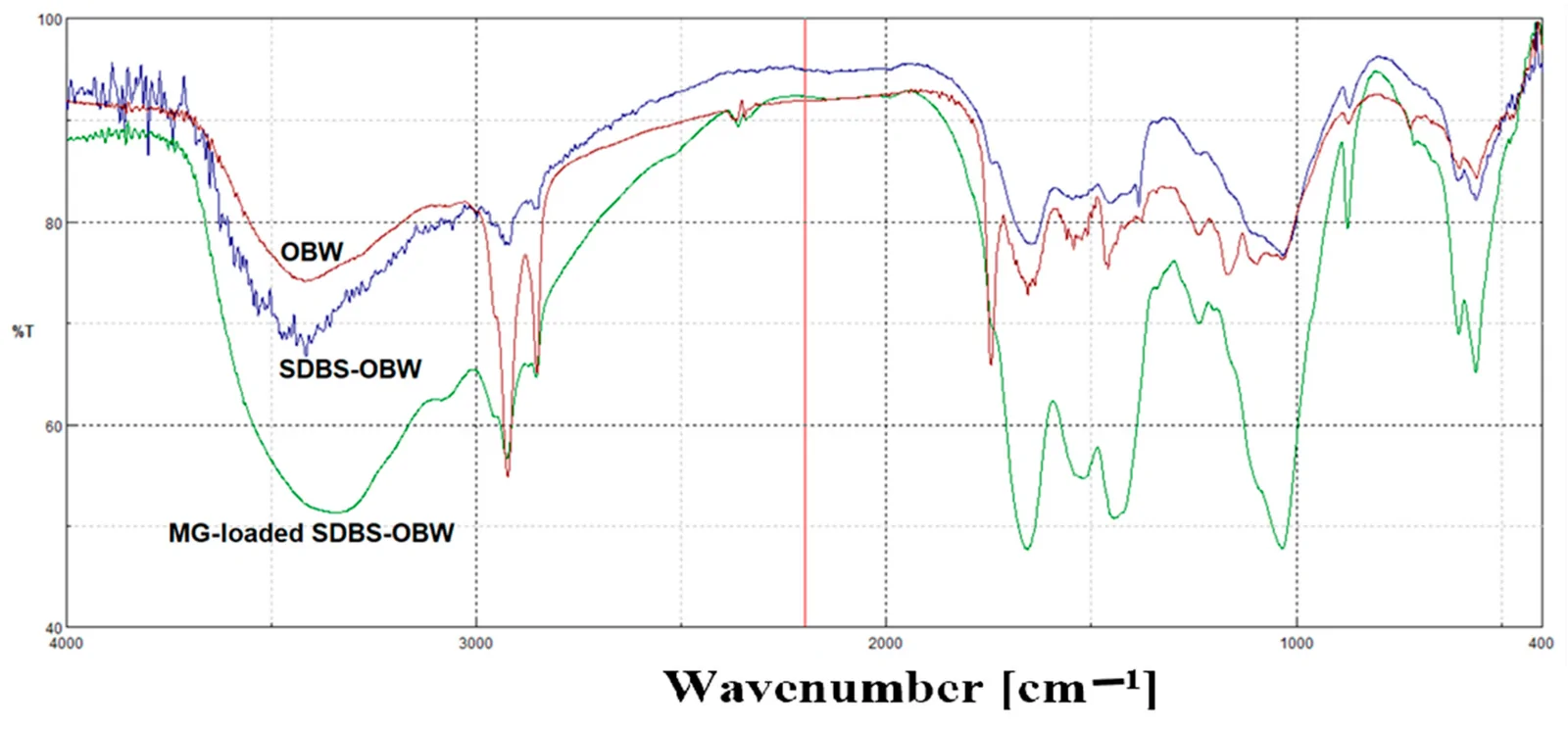
FT-IR spectra of OBW, SDBS-OBW, and MG-loaded SDBS-OBW.

The FT-IR spectra of raw OBW (red curve), SDBS-OBW (blue curve), and MG-loaded SDBS-OBW (green curve) are compared in Figure 3. The observed absorption bands, their assignments, and their phase-specific variations are summarized in Table S3. The raw OBW spectrum exhibits the characteristic absorption bands of a hydroxyapatite-rich bone mineral matrix with residual organic matter [19]. The broad band at 3300–3500 cm^−1^ is assigned to the O–H stretching vibration (ν(O–H)) of adsorbed water molecules and structural hydroxyl groups within the hydroxyapatite lattice [2]. The doublet at 2920–2850 cm^−1^ is assigned to the asymmetric and symmetric C–H stretching vibrations (ν_as_(C–H) and ν_s_(C–H)) of residual organic matter, including collagen and lipids [20]. The bands at ~1650 and ~1540 cm^−1^ are assigned to amide I (C=O stretching) and amide II (N–H bending coupled with C–N stretching) of residual collagen, respectively [2,20]. The bands at ~1450 and ~1415 cm^−1^ are assigned to the ν_3_ asymmetric stretching of carbonate (CO_3_^2−^) ions substituted into the hydroxyapatite lattice [2,20]. The strong, complex band at 1000–1100 cm^−1^ is assigned to the ν_3_ asymmetric stretching of phosphate (PO_4_^3−^), the principal mineral constituent of bone hydroxyapatite [20]. The band at ~960 cm^−1^ is assigned to the ν_1_ symmetric stretching of phosphate (PO_4_^3−^) [2,19]. The bands at ~603 and ~560 cm^−1^ are assigned to the ν_4_ bending mode of phosphate (PO_4_^3−^) [20].

After SDBS immobilization, the most prominent spectral changes are the decreases in the bands associated with collagen and organic matter. Specifically, the C–H stretching bands at 2920–2850 cm^−1^ exhibit a dramatic reduction in intensity relative to raw OBW. This reduction is attributed to the partial removal of residual organic matter (fat, tissue, and collagen) from the bone matrix during the surfactant modification process (heating at 70 °C, ethanol–water washing, and drying) [20]. No C–H band attributable to SDBS could be resolved, so FT-IR gives no unambiguous evidence for SDBS on the surface. Similarly, the amide I band at ~1650 cm^−1^ and the amide II band at ~1540 cm^−1^ both exhibit decreased intensity relative to raw OBW. This decrease is consistent with the partial removal of residual collagen during the SDBS modification process, as well as possible coverage of remaining collagen by SDBS [20]. In addition, the O–H band at 3300–3500 cm^−1^ exhibits a shift toward higher wavenumbers (from ~3350–3400 cm^−1^ to ~3400–3450 cm^−1^) together with significant broadening. This shift and broadening are attributed to: (i) partial removal of strongly hydrogen-bonded water during the modification process, which weakens the hydrogen-bonding network and shifts the O–H stretch to higher wavenumbers; and (ii) hydration of the sulfonate headgroup (–SO_3_^−^), which introduces new O–H contributions at higher wavenumbers and increases the heterogeneity of O–H environments [20]. The phosphate band at 1000–1100 cm^−1^ exhibits a slight broadening and shift, and subtle changes are observed in the low-wavenumber region at ~560–603 cm^−1^. The sulfonate bands (about 1180, 1130 and 1040 cm^−1^) are not resolved from the phosphate band, so FT-IR cannot confirm sulfonate–calcium/phosphate interaction [20]. The carbonate bands at ~1450 and ~1415 cm^−1^ remain largely unchanged, indicating that the hydroxyapatite mineral structure is preserved after SDBS modification.

After MG adsorption, the most prominent spectral changes are the increases in intensity and slight shifts in most bands. Specifically, the O–H band at 3300–3500 cm^−1^ becomes the strongest and broadest among the three samples. This enhancement is attributed to the adsorption of malachite green and associated water molecules, as well as hydrogen bonding between the dimethylamino groups (–N(CH_3_)_2_) of MG and surface O–H groups [21,22]. The amide I band at ~1650 cm^−1^ is strongly enhanced relative to SDBS-OBW. This enhancement is attributed to the overlap of the amide I band with the aromatic C=C stretching vibrations of the benzene rings and the C=N stretching vibrations of the central imine group in the malachite green molecule [20,21]. Similarly, the amide II band at ~1540 cm^−1^ is also enhanced relative to SDBS-OBW, attributed to the overlap of the amide II band with the aromatic C=C stretching vibrations of MG. The region at 1400–1600 cm^−1^ exhibits a pronounced, broad, and strong absorption, which is attributed to the aromatic C=C and C=N stretching modes of MG overlapping with the existing carbonate and amide II bands. This region is present in all three samples, but its intensity increases in the order: raw OBW < SDBS-OBW < MG-loaded SDBS-OBW. The strong enhancement in the MG-loaded sample provides direct spectroscopic evidence for MG adsorption. The phosphate band at 1000–1100 cm^−1^ shows further broadening and increased intensity, and the low-wavenumber region at ~603 and ~560 cm^−1^ exhibits strong, sharp bands. These changes are consistent with MG–surface interactions involving the phosphate groups [23]. Almost all peaks in the MG-loaded SDBS-OBW spectrum are slightly shifted and stronger relative to both raw OBW and SDBS-OBW. These shifts and intensity enhancements are attributed to MG–surface interactions involving electrostatic attraction, π–π stacking, and hydrogen bonding, which alter the local chemical environment of the surface functional groups and increase the overall IR absorption of the material [24].

**Figure 4:**
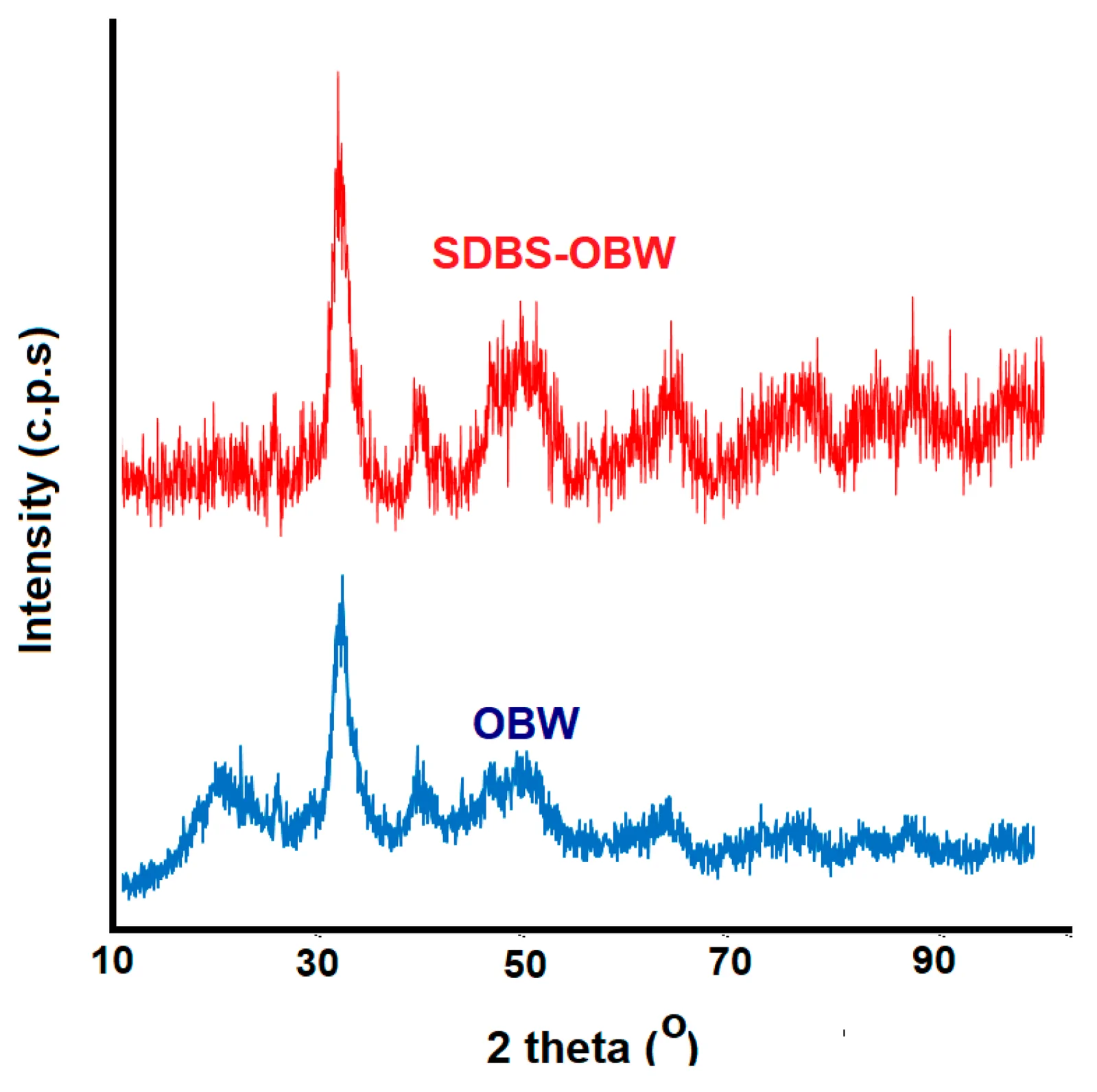
Wide-range XRD pattern of OBW and SDBS-OBW, showing the characteristic reflections of poorly crystalline calcium hydroxyapatite.

The XRD patterns of raw OBW and SDBS-OBW are compared in Figure 4. Both patterns exhibit the characteristic reflections of poorly crystalline calcium hydroxyapatite, with the main diffraction peak at approximately 32° (the 26° reflection is not resolved) and additional reflections in the 40–50° 2θ range [25]. The XRD pattern of raw OBW (blue curve) is characterized by a relatively high diffuse background and broad, poorly resolved peaks, which is consistent with a poorly crystalline hydroxyapatite phase containing a significant amount of amorphous organic matter (residual collagen, lipids, and other organic compounds) that partially obscures the mineral phase. After SDBS modification, the XRD pattern of SDBS-OBW (red curve) shows a reduced diffuse background relative to raw OBW; no change in peak sharpness was quantified. This improvement in crystallinity is attributed to the partial removal of organic matter during the SDBS modification process (heating at 70 °C, ethanol–water washing, and drying), which exposes more of the inorganic hydroxyapatite phase and reduces the amorphous background contribution. The positions of the principal hydroxyapatite reflections are preserved after SDBS modification, indicating that the mineral structure of the bone matrix is not significantly altered by the modification process. The additional reflections observed in the 40–50° 2θ range in SDBS-OBW are consistent with the improved exposure of the hydroxyapatite crystalline phase after removal of part of the organic matrix [2,20].

**Figure 5:**
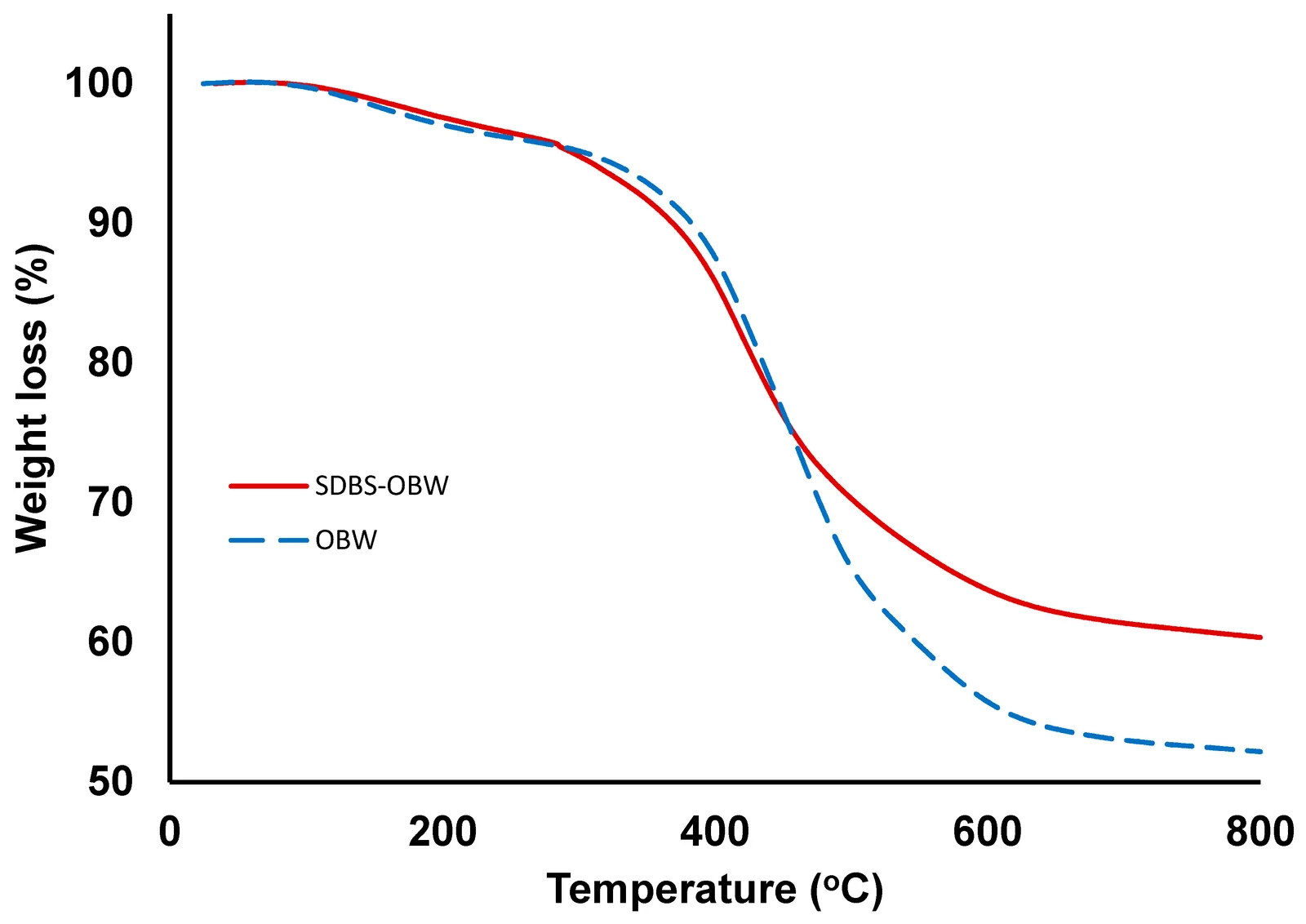
Thermogravimetric analysis (TGA) of OBW and SDBS-OBW from 25 to 800 °C under argon at a heating rate of 10 °C min^−1^.

The thermal degradation behavior of raw OBW (blue curve) and SDBS-OBW (red curve) was evaluated by thermogravimetric analysis (TGA) from 25 to 800 °C under argon at a heating rate of 10 °C min^−1^ (Figure 5). Both curves exhibit three main weight-loss stages, corresponding to dehydration, decomposition of organic matter, and decomposition of the mineral phase. The first stage (25–200 °C) corresponds to a gradual weight loss in both materials, attributed to the evaporation of adsorbed water and loosely bound moisture from the material surface [25]. In raw OBW (blue curve), the weight loss in this region is slightly higher than in SDBS-OBW, consistent with the more hydrophilic nature of the pristine bone matrix and its higher moisture content. The second stage (200–500 °C) represents the major weight-loss region for both materials. In raw OBW (blue curve), this stage exhibits a pronounced and steep decline, attributed to the decomposition of the higher organic content of the pristine bone waste, including residual collagen, lipids, and other organic matter [25]. In SDBS-OBW, the loss in this stage is lower than in raw OBW (27.3% vs. 31.7%), so any retained SDBS is far below the nominal loading (0.5–1.5 meq g^−1^, about 17–52 wt.%) [2,20]. The total weight loss in this region is higher for raw OBW than for SDBS-OBW, consistent with the partial removal of organic matter during the SDBS modification process. The third stage (500–800 °C) exhibits a more gradual weight loss in both materials, attributed to the decomposition of carbonate (CO_3_^2−^) groups substituted in the hydroxyapatite lattice and the dehydroxylation of the mineral phase [25]. The loss in this stage is 13.1% for raw OBW and 9.9% for SDBS-OBW. The final residual mass at 800 °C is lower for raw OBW (blue curve) than for SDBS-OBW (red curve), consistent with the higher organic content and lower inorganic fraction of the pristine material. After SDBS modification, the partial removal of organic matter increases the proportion of the inorganic hydroxyapatite phase, resulting in a higher residual mass in SDBS-OBW. These results are consistent with the XRD analysis, which showed a reduced amorphous background in SDBS-OBW, and with the FT-IR analysis, which showed decreased C–H and amide band intensities after SDBS modification [2,20]. Collectively, these results are consistent with partial removal of organic matter from the bone matrix, increases the proportion of the inorganic hydroxyapatite phase, and preserves the mineral structure of the material.

Overall, the characterization results are consistent with a hydroxyapatite-rich structure of SDBS-OBW and the presence of surface functionalities relevant to malachite green adsorption. Detailed characterization results and extended interpretation of the SEM–EDS, FT-IR, XRD, and TGA analyses are provided in Supplementary Material-S2.

To test the plausibility of the electrostatic mechanism, the pH of point of zero charge (pH_PZC) of raw OBW, SDBS-OBW, and MG-loaded SDBS-OBW was determined by the solid addition method, in which a fixed mass of the solid was added to electrolyte solutions pre-adjusted to different initial pH values, and the equilibrium pH was measured after equilibration. The pH_PZC_ was taken as the pH at which the curve of ΔpH (pH_final_ − pH_initial_) versus pH_initial_ intersects zero [19,26]. The measured pH_PZC_ values were 7.8 for raw OBW,6.1 for SDBS-OBW, and 8.4 for MG-loaded SDBS-OBW. The PZC of raw OBW is 7.8, which is consistent with the hydroxyapatite-rich mineral composition of the bone matrix. At pH < 7.8, the raw OBW surface is positively charged; at pH > 7.8, it is negatively charged. Because malachite green is cationic, raw OBW can only develop favorable electrostatic attraction toward MG^+^ at pH values well above 7.8, which explains its comparatively low adsorption capacity toward MG. Following SDBS modification, the PZC decreased from 7.8 to 6.1, representing a downward shift of approximately 1.7 pH units. This decrease is consistent with the introduction of negatively charged groups on the bone surface, since the immobilized SDBS molecules shift the surface charge toward more negative values across the entire investigated pH range. At pH 7.0—a representative circumneutral condition—raw OBW is nearly neutral (close to its PZC of 7.8), whereas SDBS-OBW is net negative (only 0.9 pH units above its pH_PZC of 6.1). This contrast isolates the effect of SDBS modification from other variables and demonstrates that the modification substantially increases the negative surface charge available for electrostatic attraction with cationic MG^+^. Following adsorption of malachite green, the PZC increased from 6.1 to 8.4, representing an upward shift of approximately 2.3 pH units relative to SDBS-OBW. This shift is the reverse of that induced by SDBS modification and is fully consistent with the adsorption of a cationic species onto the negatively charged surface. The adsorbed MG^+^ cations neutralize the negative sulfonate sites and contribute positively charged dimethylamino groups to the surface, thereby shifting the net surface charge toward more positive values. This reversal of the pH_PZC shift is consistent with electrostatic binding of the cationic dye to the anionic sulfonate sites introduced by SDBS modification. Malachite green is a cationic dye that forms the colourless carbinol by hydroxide addition (pK_R^+^ ≈ 6.9), so it is largely carbinol at pH 9 [27,28,29]. Under the pH range investigated in this study (2–11), MG exists predominantly as the cationic species (MG^+^) at pH < 6.9, as a mixture of MG^+^ and the neutral carbinol form at pH 6.9–10.5, and as the neutral carbinol form at pH > 10.5. The highest removal efficiency was observed at pH 7–9, which coincides with the pH range in which (i) the SDBS-OBW surface is strongly negatively charged (pH > pH_PZC = 6.1), and (ii) MG^+^ is the dominant form at pH < 6.9. This coincidence is consistent with an electrostatic attraction mechanism.

### 3.2. Identification of Key Adsorption Variables

**Figure 6:**
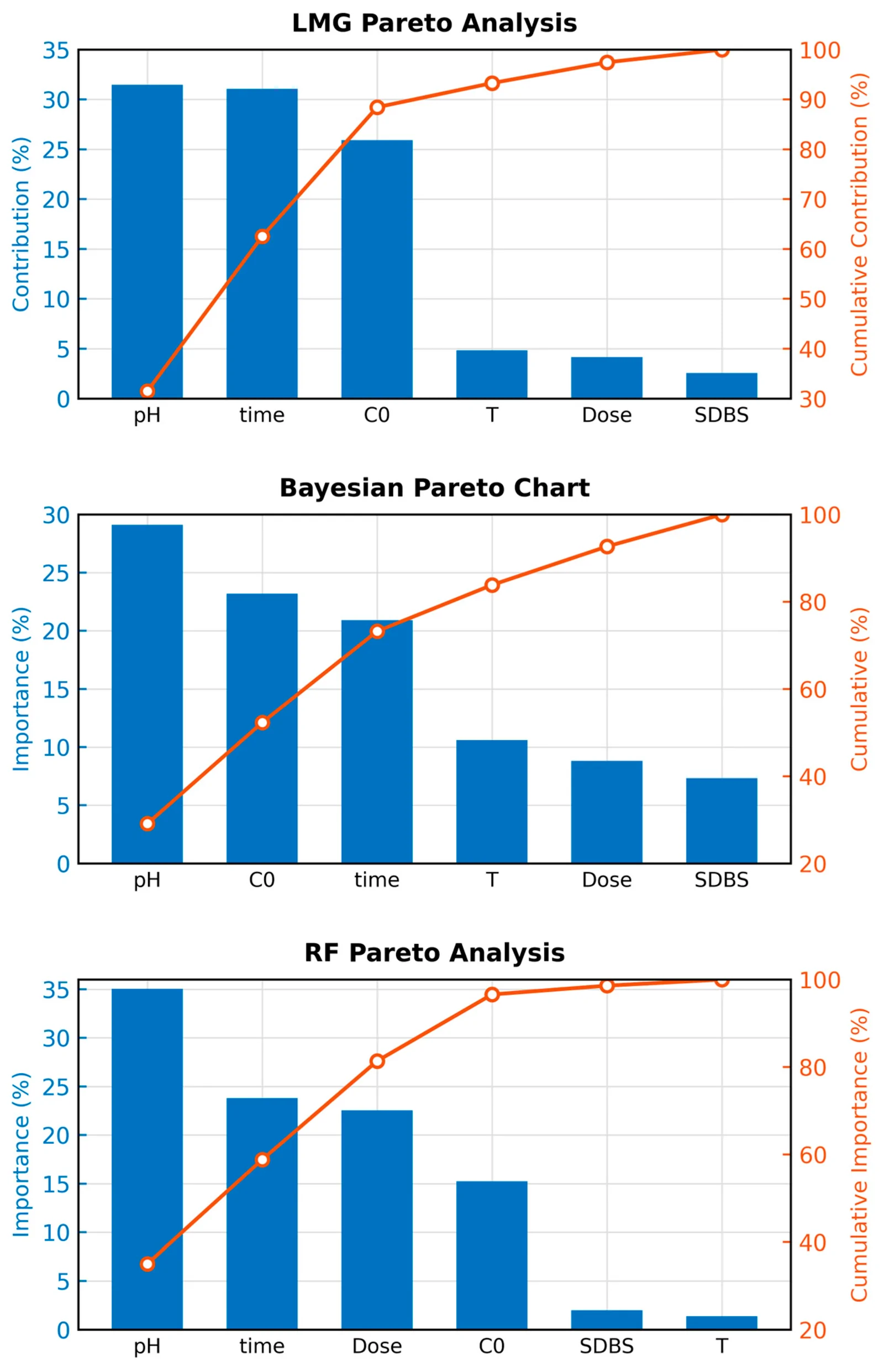
Relative importance of operational variables affecting malachite green removal efficiency according to the LMG decomposition method, posterior Bayesian coefficients, and permutation-based Random Forest analysis.

**Figure 7:**
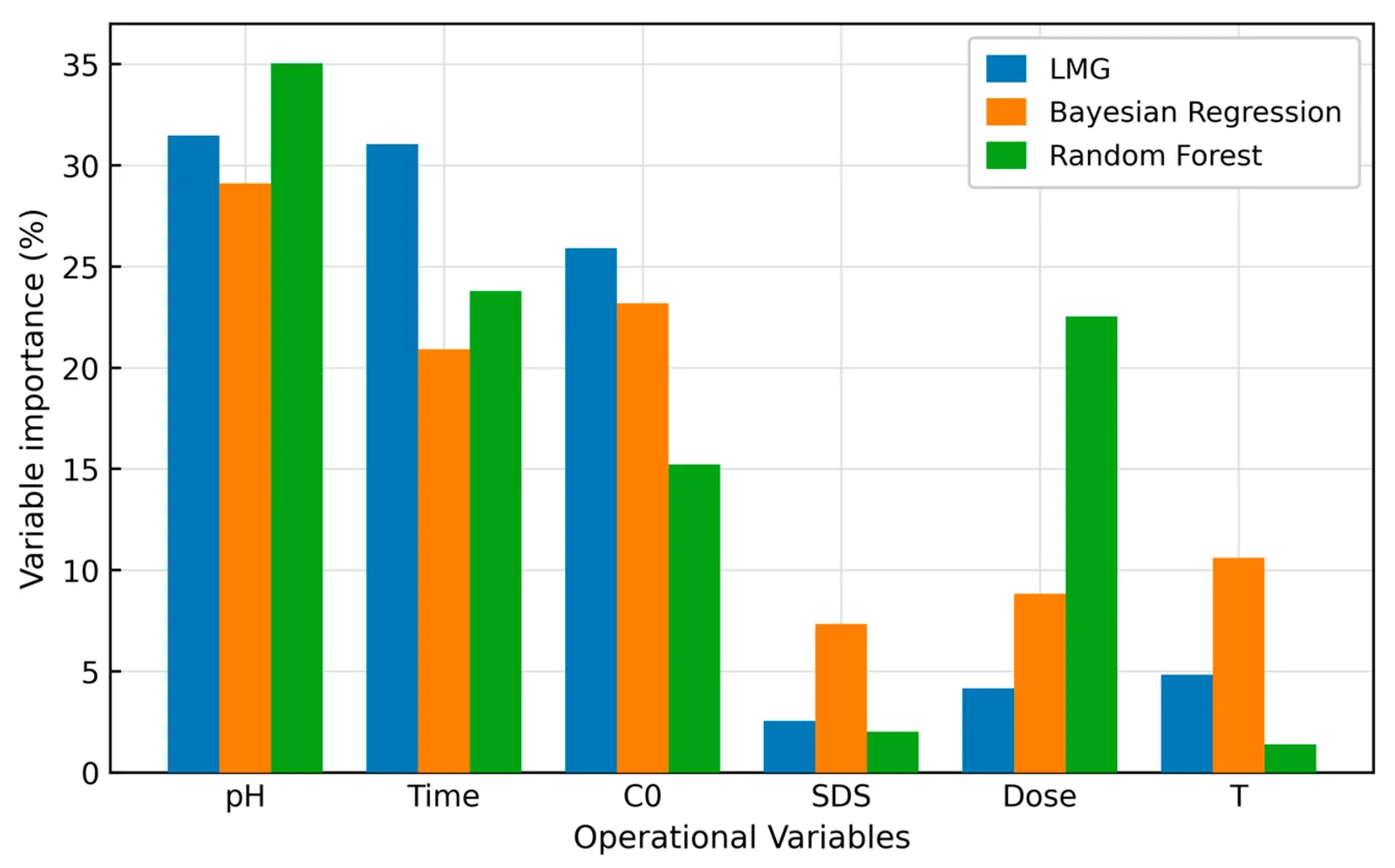
Comparison of variable importance estimated by the LMG, Bayesian regression, and Random Forest approaches.

Understanding the relative influence of operational variables is essential for elucidating the adsorption mechanism and optimizing process performance. The resulting importance rankings from three complementary approaches, the LMG method, Bayesian regression, and RF analysis, are illustrated in Figure 6, while their integrated comparison is presented in Table 1 and Figure 7.

The LMG analysis revealed that pH (31.48%), contact time (31.05%), and initial dye concentration (25.91%) were the variables that accounted for most of the explained variance in this dataset (Table 1). Collectively, these three factors accounted for approximately 88% of the total explained variance. Temperature, SDBS dosage, and adsorbent dosage were each varied over narrow or few levels (temperature in four runs; SDBS dosage in a series run at pH 2 and 55 °C), so their low importance may partly reflect the design rather than a weak physical effect. Temperature, adsorbent dosage, and SDBS concentration contributed only 4.84%, 4.17%, and 2.55%, respectively, suggesting comparatively limited influence within the investigated experimental domain.

The Bayesian regression model produced a broadly similar ranking to LMG, with pH first, but with initial concentration (23.19%) ranked above contact time (20.91%). Temperature showed a moderate influence (10.61%), whereas adsorbent dosage (8.83%) and SDBS concentration (7.34%) contributed less substantially to the overall prediction of removal efficiency (Table 1). BR and LMG are both derived from the same linear model, so their partial agreement is expected and is not independent confirmation.

The Random Forest model yielded a comparable but not identical ranking pattern (Figure 6). pH remained the most influential variable (35.04%), followed by contact time (23.79%) and adsorbent dosage (22.54%). However, unlike the Bayesian and LMG analyses, the RF model assigned substantially smaller importance to initial dye concentration (15.23%) (Table 1). The higher RF importance assigned to adsorbent dosage may reflect nonlinear dependencies or interactions involving dosage that are not represented by the linear BR formulation; however, the present modeling analysis does not independently establish the underlying physical interaction mechanism. In adsorption systems, dosage can influence the availability of active adsorption sites, particle aggregation, and mass-transfer phenomena simultaneously; therefore, its effect may become evident only when nonlinear dependencies are considered [30,31].

To facilitate direct comparison among the three modeling approaches, the relative importance values were consolidated into a single comparative framework (Figure 7). Despite methodological differences, all three approaches ranked pH first. Contact time was second in LMG and RF but third in BR. pH dominance was therefore the only result robust to the choice of method, whereas the ranking of the remaining predictors depended on the method.

From a mechanistic perspective, the dominance of pH can be attributed to its direct influence on adsorbent surface charge and dye speciation. Malachite green is a cationic dye whose adsorption behavior is strongly dependent on electrostatic interactions. Variations in solution pH alter the ionization state of functional groups on the SDBS-modified ostrich bone surface and consequently affect the adsorption affinity toward dye molecules. The strong contribution of contact time confirms that adsorption kinetics play a critical role in determining removal efficiency, whereas the importance of initial concentration reflects the effect of concentration gradients and mass-transfer driving forces on adsorption capacity [32].

The comparatively low importance attributed to SDBS dosage by all three modeling approaches (2.55% by LMG, 7.34% by BR, and 2.01% by RF) should not be misinterpreted as evidence that SDBS modification is unnecessary. Rather, the SDBS-dosage series was measured at pH 2 and 55 °C, where electrostatic attraction between the sulfonate groups and MG^+^ is expected to be least favourable (Section 3.1). The low importance of SDBS dosage therefore cannot be taken as evidence that surfactant loading is unimportant at the pH values where adsorption is high (pH 7–9). This result should not be interpreted as evidence that SDBS modification itself is unnecessary; instead, it indicates that the response was less sensitive to changes in SDBS dosage within the experimental range examined. The modification process is therefore essential for creating the negatively charged surface required for electrostatic attraction with cationic MG^+^. This distinction between the necessity of modification and the sensitivity of performance to modifier dosage is critical for correct interpretation of the variable-importance results. Likewise, the limited contribution of temperature indicates that adsorption performance was relatively insensitive to thermal fluctuations within the studied interval. Collectively, these findings demonstrate that within the sampled design, pH, contact time, and initial concentration explained most of the variance. A factorial or response-surface design is needed to establish which variables should be prioritized for optimization.

The convergence of conclusions obtained from three fundamentally different analytical approaches indicates that the dominance of pH is robust to method choice within this dataset, but it does not by itself validate the proposed mechanism and establishes a robust basis for subsequent model-performance evaluation and uncertainty analysis.

### 3.3. Bayesian Interpretation of Predictor Effects

While the comparative importance analysis presented previously identified the dominant operational variables governing malachite green removal, BR provides additional insight into the direction, magnitude, and uncertainty associated with each predictor. Unlike the LMG and RF approaches, Bayesian inference estimates full posterior distributions for model parameters, thereby allowing direct assessment of parameter uncertainty and statistical credibility.

**Figure 8:**
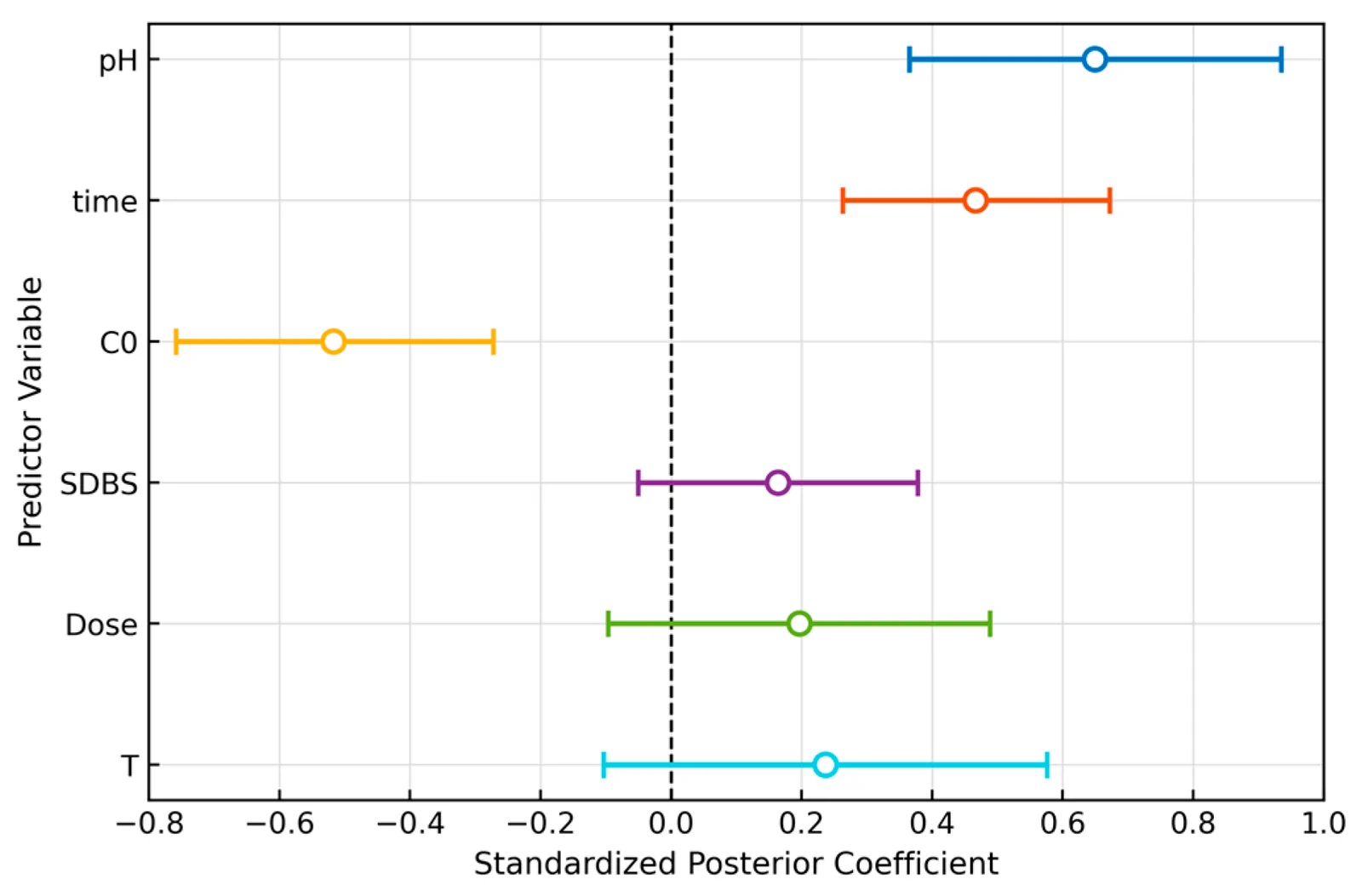
Posterior mean coefficients and 95% credible intervals obtained from Bayesian regression analysis for the prediction of malachite green removal efficiency.

The posterior coefficient estimates and their corresponding 95% credible intervals are summarized in Table 2 and visually represented in Figure 8. The forest plot illustrates both the magnitude of the posterior mean coefficients and the associated uncertainty intervals, enabling rapid identification of variables exerting statistically meaningful effects on adsorption performance.

Inspection of Figure 8 reveals that pH exhibited the largest positive posterior coefficient among all investigated variables, confirming its dominant role in controlling adsorption efficiency. The 95% CrI for pH (0.365–0.935) lies entirely above zero, indicating a credibly positive effect, although its width (0.57) is comparable to those of temperature and adsorbent dosage. This observation is consistent with the importance rankings reported previously and supports the well-established role of pH in regulating adsorbent surface charge and electrostatic interactions between malachite green molecules and adsorption sites [33].

Initial dye concentration and contact time also displayed substantial posterior coefficients, further confirming their importance in determining adsorption performance. Contact time showed a positive posterior coefficient (β = 0.467), whereas initial MG concentration showed a negative coefficient (β = −0.517), indicating that removal efficiency tended to increase with contact time but decrease with increasing initial concentration after accounting for the other predictors.

The 95% credible intervals of temperature (−0.103 to 0.576), adsorbent dosage (−0.096 to 0.488), and SDBS dosage (−0.051 to 0.378) all include zero, so the direction of their effects cannot be established from these data. The wider uncertainty bands observed for these parameters suggest greater variability in their estimated effects and indicate that their independent contributions to adsorption performance are less pronounced than those of pH, contact time, and initial concentration. In particular, the relatively broad credible intervals associated with SDBS dosage imply that variations in surfactant concentration within the investigated range produced only limited improvements in adsorption efficiency once surface modification had been successfully established [34].

The posterior statistics presented in Table 2 provide additional evidence regarding the reliability of parameter estimates. Variables characterized by relatively narrow credible intervals can be interpreted with greater confidence, whereas wider intervals indicate increased uncertainty and reduced precision. Consequently, pH, contact time, and initial concentration not only emerged as the most influential variables in the importance analysis but also demonstrated the most stable and reliable posterior estimates.

All parameters satisfied a priori convergence criteria, with split $\hat{R}$ ≤ 1.0001 and bulk ESS values between approximately 3100 and 40,000; the trace plots showed stationary, well-mixed chains without trend or drift (Figure S1; Table 2). In the posterior-predictive checks, the replicated data sets reproduced the dispersion and range of the observed removal efficiencies (p_SD = 0.68, p_min = 0.32, p_max = 0.99), and the 95% posterior-predictive intervals covered 96.2% of the observations (2 of 52 runs outside), indicating no systematic lack of fit of the Gaussian residual structure (Figure S2; Table S1). On the standardised scale, the posterior residual variance (σ^2^ = 0.511, 95% CrI 0.335–0.774) corresponds to a posterior R^2^ of 1 − σ^2^/Var(y) = 0.489 (95% CrI 0.226–0.665), the probabilistic counterpart of the coefficient of determination for the fitted model (Table 2).

From a mechanistic perspective, the Bayesian results reinforce the conclusion that adsorption performance is governed primarily by physicochemical interactions occurring at the adsorbent–solution interface. The dominant role of pH highlights the importance of surface-charge regulation and electrostatic attraction mechanisms, whereas the significant effects of contact time and dye concentration emphasize the contributions of adsorption kinetics and mass-transfer processes. The comparatively lower certainty associated with SDBS dosage and adsorbent dosage suggests that these variables act primarily as secondary modifiers rather than principal controlling factors under the investigated experimental conditions.

A major advantage of the Bayesian framework is its ability to explicitly quantify uncertainty in parameter estimation. Rather than producing a single deterministic coefficient, Bayesian regression provides a probability distribution for each predictor effect, thereby allowing a more realistic representation of the inherent variability associated with adsorption systems. This capability is particularly valuable for environmental applications, where experimental variability and process uncertainty often influence model reliability and decision-making.

Overall, the Bayesian analysis is consistent with the variable-importance findings presented previously while providing additional information on the direction and uncertainty of predictor effects. The posterior coefficient estimates agree with the importance rankings and show that pH, contact time and initial dye concentration were the variables with credibly non-zero effects on removal within this dataset. This is consistent with a surface-charge-dependent mechanism, which is also consistent with the pH_PZC results (Section 3.1); the regression itself does not establish the mechanism.

### 3.4. Predictive Performance of Machine Learning Models

The predictive capabilities of the developed machine-learning models were evaluated using BR and RF approaches. Unlike the LMG method, which primarily quantifies relative variable importance, both BR and RF can be employed for prediction of malachite green removal efficiency under different operating conditions.

**Figure 9:**
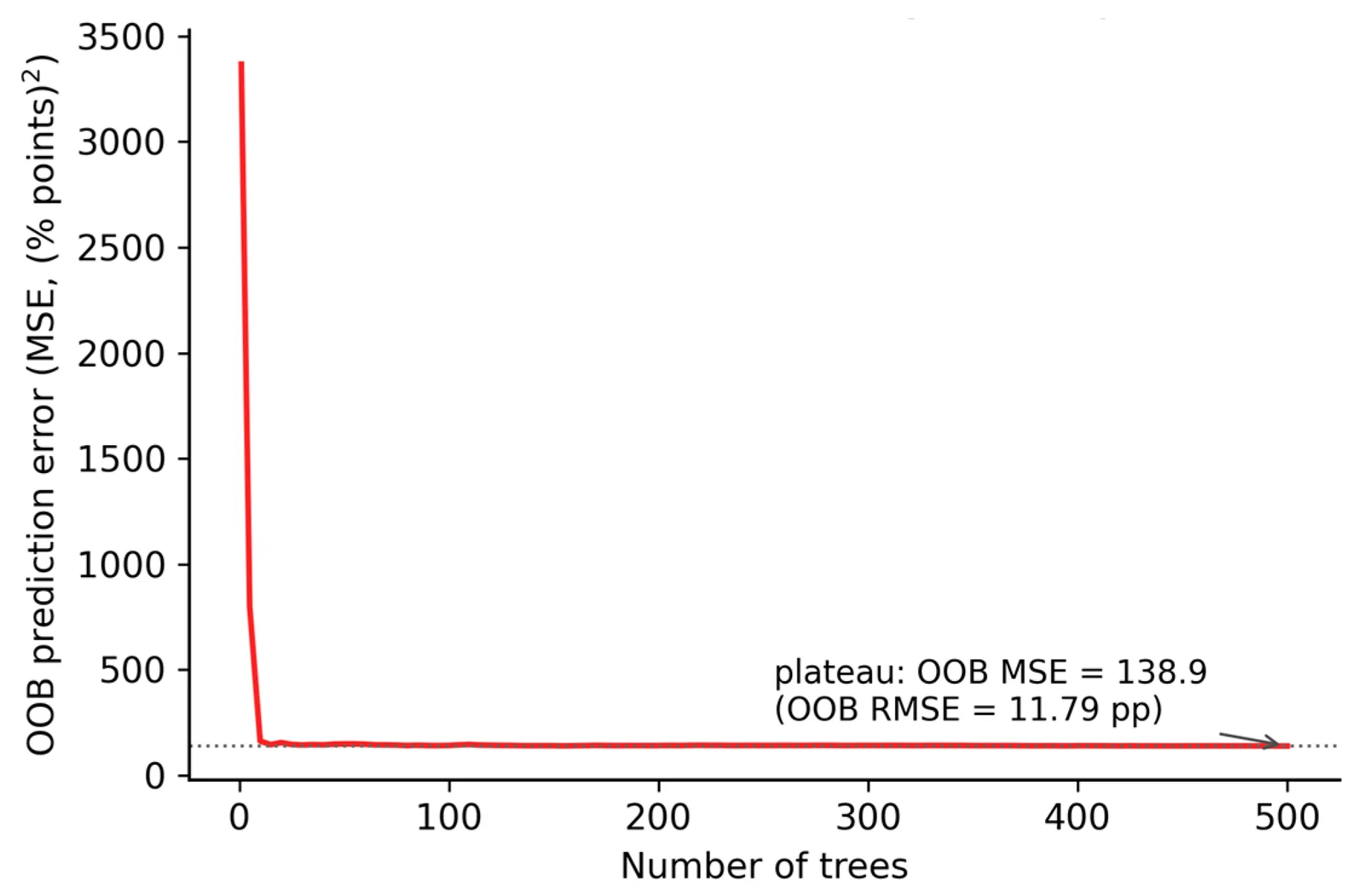
Out-of-bag (OOB) prediction error as a function of the number of trees in the RF model (n_estimators = 500, max_features = 2, random_state = 42). The OOB error stabilises beyond approximately 100 trees and reaches 138.9 (percentage points)^2^ at 500 trees, equivalent to an OOB RMSE of 11.79 pp.

The predictive performance of the RF model was first evaluated through out-of-bag (OOB) error analysis (Figure 9). The OOB error decreased rapidly during the initial stages of ensemble construction, stabilised beyond approximately 100 trees, and approached a final OOB mean squared error of 138.9 (percentage points)^2^ at 500 trees, equivalent to an OOB RMSE of 11.79 pp. The convergence of the OOB error curve confirms that a sufficient number of decision trees were incorporated into the ensemble and that additional trees contribute only marginal improvements. The corresponding OOB coefficient of determination was R^2^ = 0.822, with an OOB MAE of 7.85 pp (Table 3). Because each observation is predicted exclusively by the trees whose bootstrap sample excluded it, OOB provides an internal, row-wise estimate of prediction error. Because runs within a series are serially correlated and different series share variable levels, this estimate is optimistic (see the group-aware results below) and should be read as interpolation skill within the sampled design, not as generalisation.

**Figure 10:**
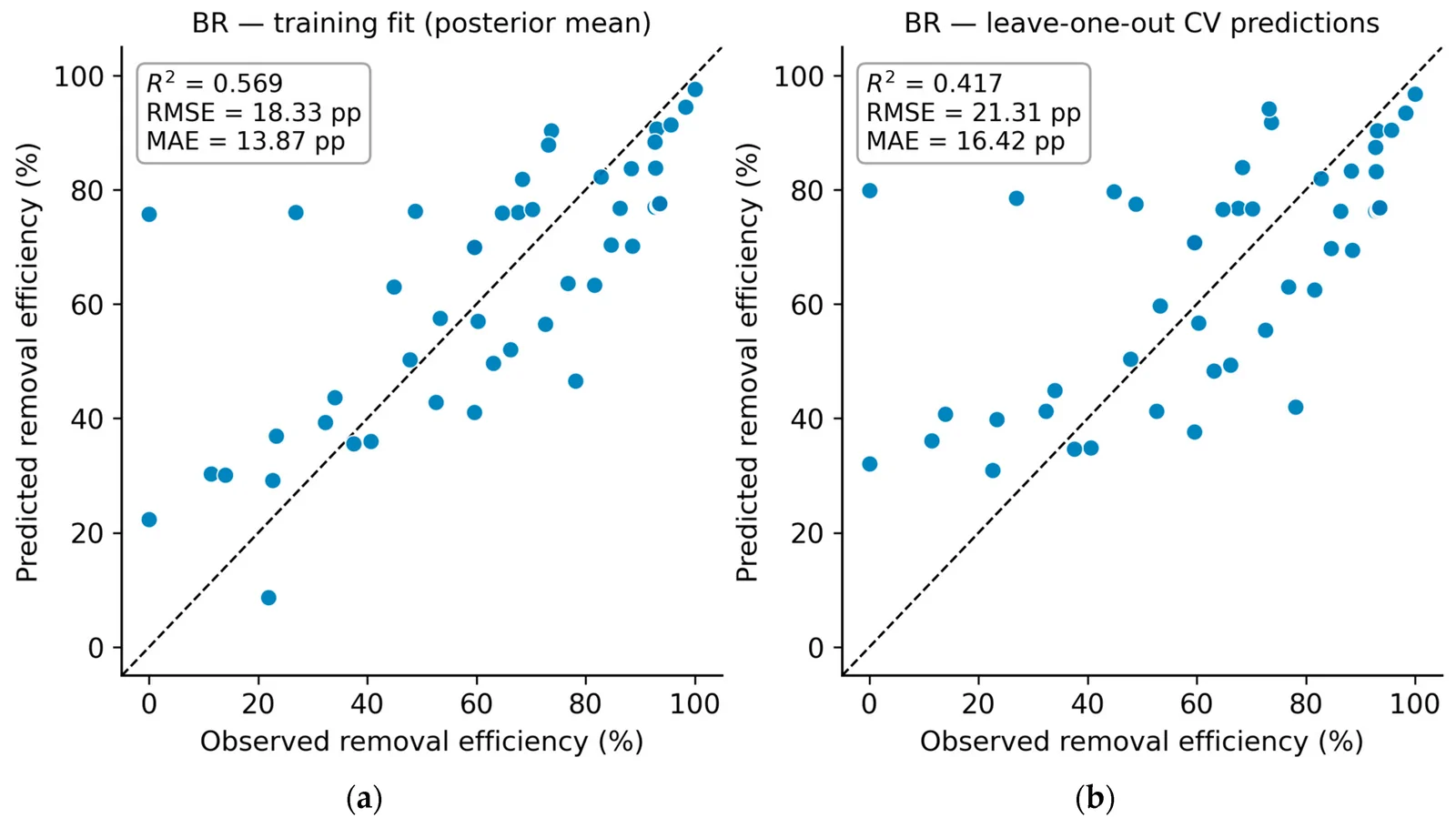
Observed versus predicted malachite green removal efficiencies obtained using the BR model: (**a**) in-bag training predictions; (**b**) leave-one-out cross-validation predictions. The dashed line indicates perfect agreement between measured and predicted values.

**Figure 11:**
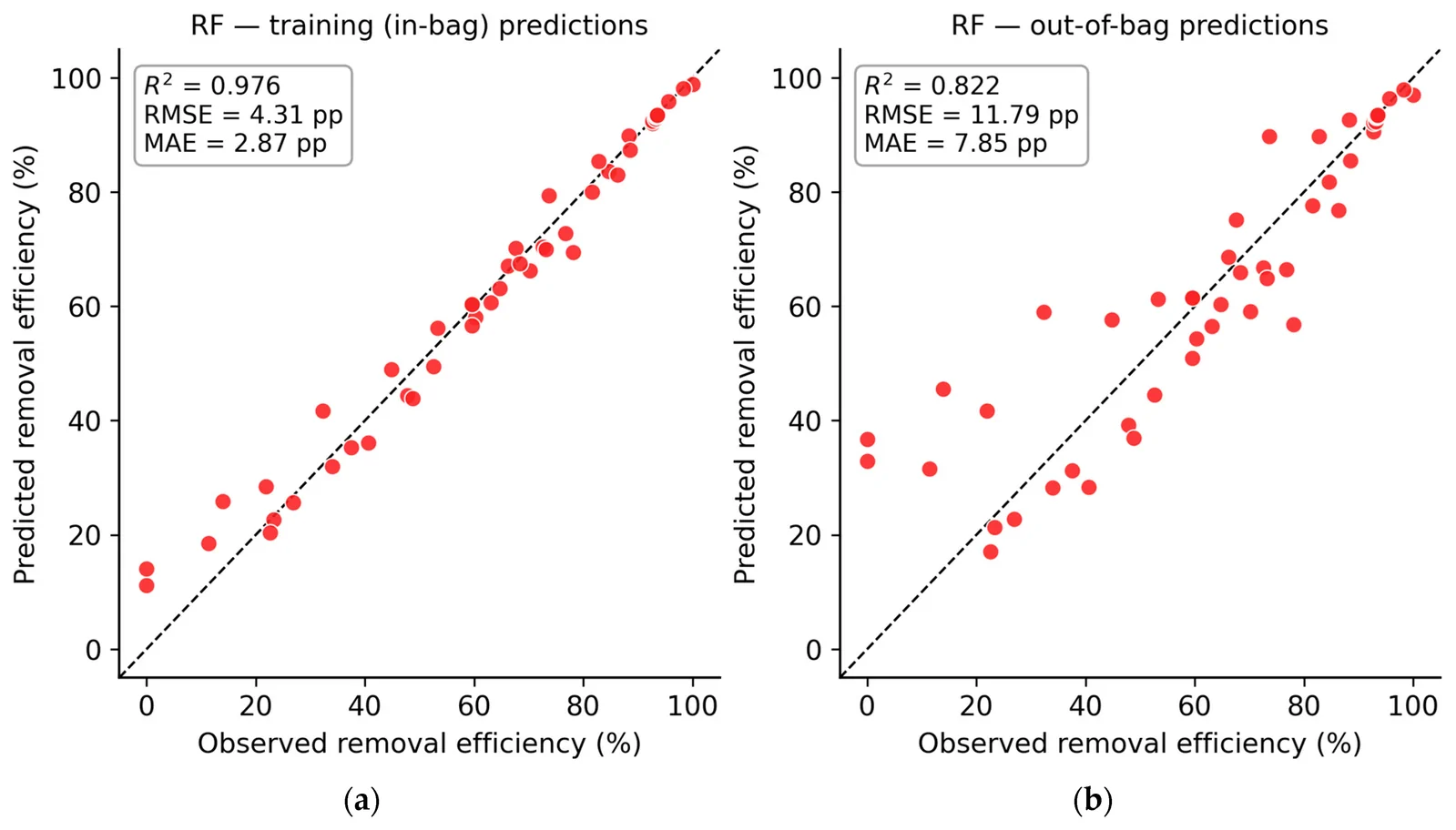
Observed versus predicted malachite green removal efficiencies obtained using the RF model: (**a**) in-bag training predictions; (**b**) out-of-bag predictions. The dashed line indicates perfect agreement between measured and predicted values.

The observed-versus-predicted relationships are presented separately for the training and the out-of-sample predictions of each model (Figure 10 and Figure 11). The in-bag training fits (BR: R^2^ = 0.569, RMSE = 18.33 pp, MAE = 13.87 pp; RF: R^2^ = 0.976, RMSE = 4.31 pp, MAE = 2.87 pp) characterise the flexibility of the two model classes but are reported for reference only, as they do not measure predictive performance (Table 3). Under genuine out-of-sample conditions, the leave-one-out cross-validated BR model achieved R^2^ = 0.417, RMSE = 21.31 pp and MAE = 16.42 pp (Figure 10b), whereas the RF model maintained R^2^ = 0.822, RMSE = 11.79 pp and MAE = 7.85 pp under OOB prediction (Figure 11b; Table 3). The tighter clustering of the OOB predictions around the 1:1 line is consistent with the flexibility of the ensemble, but it may partly reflect serial correlation between runs of the same series rather than genuine nonlinear structure. OOB error is also known to be biased under certain input structures [35].

Because the 52 runs originate from the six one-variable-at-a-time experimental series with strong serial correlation within each series, row-wise resampling schemes are expected to be optimistic, and this was confirmed quantitatively. Under naive row-wise 10-fold cross-validation (50 repeats), the RF model attained R^2^ = 0.793 ± 0.028 (RMSE = 12.69 pp) and the BR model R^2^ = 0.410 ± 0.034 (RMSE = 21.43 pp), while under leave-one-out cross-validation the corresponding values were R^2^ = 0.819 (RMSE = 11.89 pp) for RF and R^2^ = 0.417 (RMSE = 21.31 pp) for BR (Table 3). Under group-aware validation, in which entire experimental series were held out, performance declined markedly: pooled leave-one-series-out cross-validation yielded R^2^ = −0.382 (RMSE = 32.81 pp, MAE = 22.12 pp) for RF and R^2^ = −124.89 (RMSE = 313.21 pp, MAE = 123.27 pp) for BR, and repeated group-aware 3-fold CV with random series-to-fold assignment (300 repeats) gave R^2^ = −0.396 ± 0.084 (RMSE = 32.97 ± 0.98 pp) for RF (Table 3; Figure S3). The systematic gap between the row-wise and group-aware schemes demonstrates that randomised row resampling overestimates model performance when highly correlated runs of the same experimental sequence are shared between the training and evaluation sets; the group-aware results are therefore adopted as the honest estimates of predictive skill.

As an example of a single-series rotation, the entire pH series (10 runs spanning pH 2–11) was held out and predicted by models trained on the other five series (42 runs; no rows shared). The BR model reproduced the pH trend with R^2^ = 0.410 (RMSE = 21.00 pp, MAE = 17.43 pp), whereas the RF model did not generalise to this fully held-out series (R^2^ = −0.911, RMSE = 37.79 pp, MAE = 29.61 pp) (Table 3). The five remaining single-series rotations are reported in Table S4 and confirm that extrapolation to operating regions absent from the training data remains challenging for both models—a limitation attributable to the one-variable-at-a-time coverage of the experimental design rather than to either algorithm (Table S4).

A quantitative comparison of model performance under all validation schemes is summarised in Table 3. Under internal out-of-sample validation, the RF model outperformed the BR model (OOB: R^2^ = 0.822, RMSE = 11.79 pp, MAE = 7.85 pp versus LOOCV: R^2^ = 0.417, RMSE = 21.31 pp, MAE = 16.42 pp), consistent with the ability of the ensemble-learning architecture to capture complex nonlinear relationships between operational variables and adsorption performance. The group-aware results indicate that predictive performance declines substantially when entire experimental series are held out, demonstrating that the present OVAT design provides limited support for generalization across operating regions that are not represented in the training data (Table S4; Figure S3).

The difference in out-of-sample predictive performance between the two approaches highlights the inherent complexity of the adsorption process. Under internal validation the RF algorithm demonstrated greater flexibility in modelling the nonlinear physicochemical interactions associated with dye adsorption onto SDBS-modified ostrich bone waste, whereas the linear Bayesian framework remained restricted to first-order effects. The higher row-wise accuracy of RF is consistent with its ability to represent its ensemble-learning architecture, which effectively captures nonlinear relationships, high-order interactions, and complex dependencies among operational variables that are often difficult to represent using conventional regression models. Similar observations have been reported in previous studies. Breiman [9] demonstrated that Random Forest achieves high predictive accuracy and robustness through the aggregation of multiple decision trees, particularly in systems characterized by complex variable interactions. Likewise, Hastie et al. [36] reported that ensemble-learning methods frequently outperform traditional linear statistical models when nonlinear predictor–response relationships exist. In environmental and water-related applications, Mosavi et al. [37] showed that Random Forest consistently provides reliable predictions due to its ability to model complex multivariate systems while maintaining resistance to overfitting. Similarly, Yaseen et al. [38] concluded that machine-learning approaches generally outperform conventional regression methods in environmental prediction problems because they can effectively capture nonlinear responses and hidden interactions among explanatory variables. Under row-wise internal validation, RF outperformed BR. Under group-aware validation, neither model achieved positive skill overall, so the RF advantage is confined to interpolation within the sampled series. The row-wise advantage is therefore not evidence of nonlinear physical interactions, and may partly reflect RF recognising series membership from combinations of dose and C_0_.

BR and RF provide complementary information rather than a universally superior predictive model. BR offers interpretable coefficient estimates and credible intervals that describe the direction and uncertainty of predictor effects, whereas RF can represent nonlinear relationships and interactions that are not explicitly included in the linear Bayesian formulation. The RF model showed stronger internal predictive performance based on its OOB evaluation, but its performance declined substantially under group-aware and independent-series validation. Accordingly, the present results support the use of RF primarily for nonlinear pattern characterization within the investigated experimental domain, while BR provides interpretable probabilistic estimates of predictor effects. Neither model should be regarded as independently validated for extrapolation beyond the experimental series and operating regions represented in the dataset.

In the one-variable-at-a-time design, adsorbent dosage and initial concentration take only a few distinct values (0.1 and 1.0 g L^−1^; 20 and 50 mg L^−1^ outside their own series), so the combination of these variables can identify the experimental series. Row-wise validation and OOB prediction may therefore reward series recognition rather than physical learning. This is consistent with the large gap between OOB R^2^ (0.822) and leave-one-series-out R^2^ (−0.382).

### 3.5. Proposed Mechanism of Malachite Green Removal by SDBS-Modified OBW

**Figure 12:**
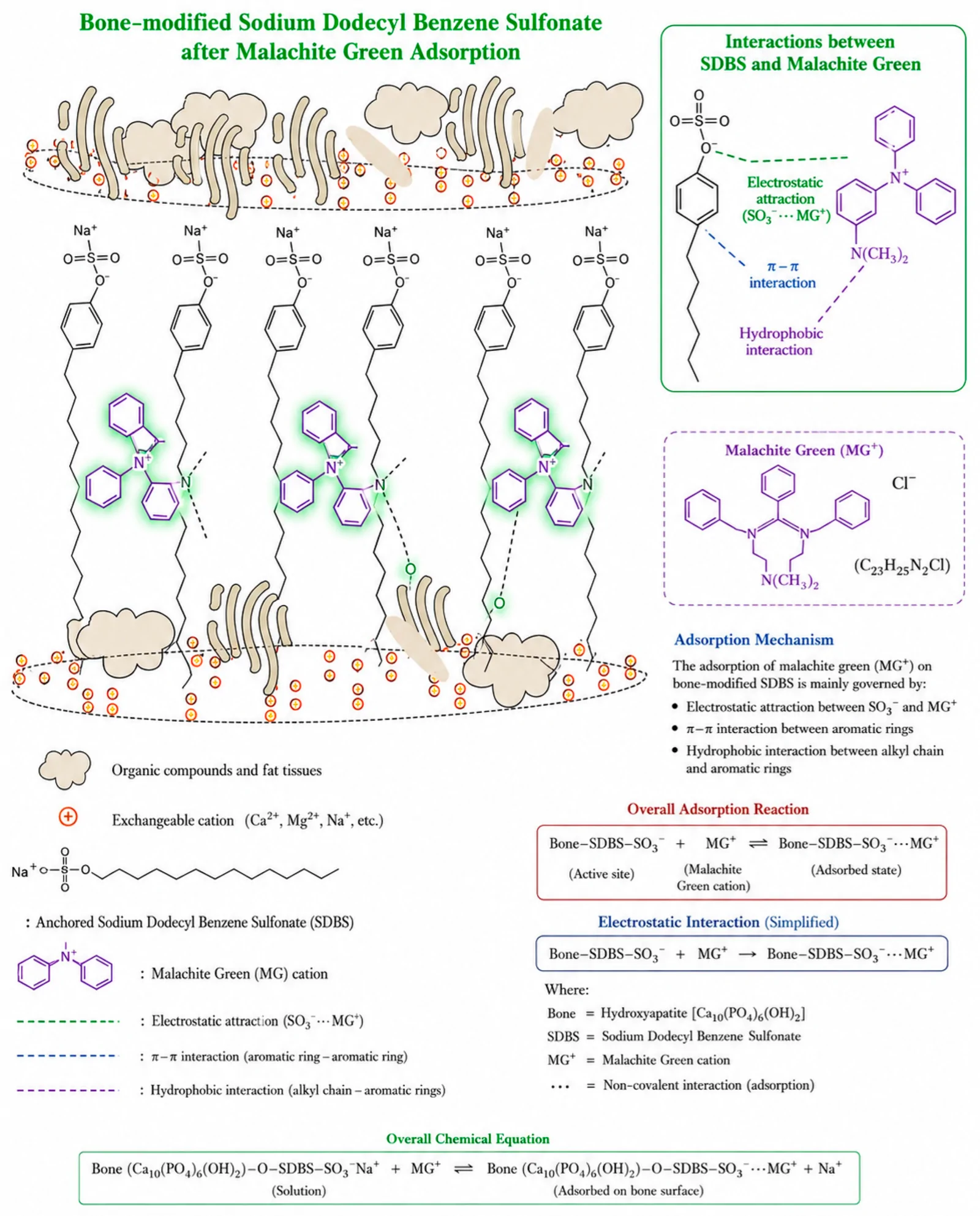
Proposed multimechanistic pathway for MG adsorption onto SDBS-modified ostrich bone waste (SDBS-OBW), illustrating electrostatic attraction between negatively charged sulfonate groups and cationic MG, together with possible π–π and hydrophobic interactions involving the aromatic and alkyl groups of SDBS.

The adsorption mechanism of malachite green by SDBS-OBW is proposed to involve multiple complementary interactions, including electrostatic attraction, π–π interactions, hydrophobic interactions, and surface-site occupation (Figure 12). The SEM–EDS, FT-IR, and XRD results confirmed a heterogeneous, hydroxyapatite-rich surface containing Ca and P as major elements, together with sulfur that is compatible with, but does not prove, SDBS retention. These characteristics provide both mineral and organic adsorption sites for interaction with the cationic MG molecule.

Electrostatic attraction is considered a major contribution because the sulfonate groups introduced by SDBS provide negatively charged sites that can attract cationic MG. This interpretation is consistent with the modeling results, in which pH was identified as one of the most influential variables, while Bayesian regression showed a positive posterior coefficient for pH (β = 0.650). The strong effect of pH is consistent with a surface-charge-dependent mechanism, which is also consistent with the pH_PZC results (Section 3.1). Similar electrostatic contributions have been reported for MG adsorption onto functionalized coal and other modified adsorbents [21,39].

The aromatic benzene group and long alkyl chain of SDBS can additionally contribute to MG retention through π–π and hydrophobic interactions, respectively. These interactions may stabilize MG molecules after their initial attraction toward sulfonate sites. Similar multiple-interaction mechanisms involving electrostatic attraction, π–π interactions, and hydrophobic effects have been reported for MG adsorption on functionalized lignin and other modified adsorbents [23,24,40].

The modeling results further support a process involving progressive occupation of heterogeneous adsorption sites. Contact time showed a positive Bayesian coefficient (β = 0.467), indicating improved removal with increasing contact time, whereas initial MG concentration showed a negative coefficient (β = −0.517), consistent with progressive occupation of a finite number of adsorption sites at higher dye concentrations. The RF importance results suggest possible nonlinear dependence (for example, for adsorbent dosage), but the one-variable-at-a-time design does not allow this to be separated from series-specific effects.

In general, the characterization and three complementary modeling approaches support a multimechanistic adsorption process dominated by surface-charge-dependent attraction and assisted by aromatic/hydrophobic interactions and progressive site occupation. However, π–π and hydrophobic interactions should be regarded as proposed mechanisms rather than directly proven molecular interactions. The mechanism illustrated in Figure 12 therefore integrates the experimentally characterized surface chemistry with the statistically identified controlling variables.

### 3.6. Implications for Adsorption Process Optimization

The integration of LMG, Bayesian regression, and Random Forest analyses provides a comprehensive framework for understanding and optimizing malachite green adsorption onto SDBS-modified ostrich bone waste. Rather than relying on a single modeling approach, the combined methodology enables simultaneous assessment of variable importance, parameter uncertainty, and predictive performance, thereby providing a more robust basis for process optimization and practical implementation.

The comparative importance analysis presented in Table 1 and Figure 7 demonstrated remarkable consistency among the three modeling approaches regarding the identification of the principal adsorption variables. Despite differences in their mathematical foundations, all three approaches ranked pH first, with contact time second in LMG and RF and third in BR, while initial dye concentration was highly ranked by LMG and BR but ranked fourth by RF. Thus, the methods showed broad agreement on the primary role of pH and contact time, with some methodological differences in the relative importance of the remaining predictors. This agreement substantially increases confidence in the robustness of the pH effect and suggests that future optimization efforts should focus primarily on these variables.

The dominant influence of pH identified by all models indicates that optimization strategies should prioritize precise control of solution acidity and alkalinity. From an adsorption perspective, pH directly governs surface charge development, protonation–deprotonation equilibria, and electrostatic interactions between malachite green molecules and active adsorption sites [33]. Consequently, even relatively small deviations from the optimal pH range may significantly reduce adsorption efficiency. The consistent ranking of pH as the most influential variable therefore highlights its importance as the primary operational control parameter during process design and industrial implementation.

Contact time emerged as the second major process variable in most analyses. This finding suggests that adsorption performance is strongly influenced by the progression of mass transfer and adsorption kinetics. The results indicate that sufficient residence time must be provided to ensure effective occupation of available adsorption sites and approach equilibrium conditions. From an engineering perspective, this observation has direct implications for reactor sizing, hydraulic retention time, and process throughput. Excessively short contact times may compromise treatment efficiency, whereas excessively long contact times may reduce process productivity without providing substantial additional removal.

Initial dye concentration was also consistently identified as a key determinant of adsorption performance. Higher concentrations increase the mass-transfer driving force and enhance the probability of interaction between dye molecules and adsorption sites. However, elevated concentrations may also accelerate site saturation and reduce removal efficiency when adsorption capacity becomes limiting. Therefore, practical process optimization should carefully balance influent concentration, adsorbent dosage, and hydraulic residence time to maintain high removal performance under varying wastewater conditions.

Bayesian regression provided additional insight into parameter uncertainty through posterior coefficient estimates and credible intervals (Table 2 and Figure 8). The relatively narrow credible intervals associated with pH, contact time, and initial dye concentration indicate that these variables exert stable and reliable effects across the investigated experimental domain. Such consistency is highly desirable for industrial applications because it suggests that process performance can be predicted with greater confidence under different operating conditions.

The predictive evaluation based on source-labelled validation demonstrated the complementary roles of the two model classes. While BR successfully quantified parameter effects and their uncertainty, the RF model achieved higher row-wise predictive performance under internal out-of-sample validation (out-of-bag R^2^ = 0.822, RMSE = 11.79 pp, versus a leave-one-out R^2^ = 0.417 and RMSE = 21.31 pp for the Bayesian model), which is suggestive of, but does not establish, nonlinear behaviour. Group-aware validation further revealed the limits of extrapolation inherent to the one-variable-at-a-time design, so that the predictive models are recommended for interpolation and scenario analysis within the investigated experimental domain, whereas experiments explicitly replicated across independent operating conditions are identified as the required next step for extrapolative prediction.

From a practical perspective, the combined modeling results suggest that different analytical tools can be employed at different stages of process development. LMG analysis is particularly useful for identifying influential variables and establishing process priorities. Bayesian regression provides mechanistic interpretation and uncertainty quantification, thereby improving scientific understanding of adsorption behavior. Random Forest can describe nonlinear patterns within the sampled experimental series. Before use for optimization or forecasting, it requires validation on independently designed experiments (e.g., a factorial or central composite design).

The integration of these complementary approaches represents a powerful strategy for adsorption-system analysis because it combines interpretability with predictive capability. Such a framework can assist researchers and engineers in designing more efficient treatment systems, reducing experimental effort, and improving process reliability. Moreover, the methodology developed in the present study can be readily extended to other dye-removal systems, adsorbent materials, and wastewater-treatment applications where multiple interacting variables influence adsorption performance.

Overall, the combined findings indicate that SDBS-modified ostrich bone waste shows substantial potential as an effective adsorbent for malachite green removal, while the integrated LMG–BR–RF framework provides a transparent workflow for adsorption-process evaluation and prediction within the investigated batch domain, although its transferability to other systems and conditions remains to be established.

## 4. Conclusions

This study investigated the adsorption of MG dye onto SDBS-OBW using an integrated modeling framework consisting of LMG, BR, and RF modeling. The combined approach enabled evaluation of variable importance, parameter uncertainty, and predictive performance within the investigated experimental domain, thereby providing more comprehensive insight into the adsorption process than a single analytical technique. The comparative analyses showed that pH was the most influential predictor in all three approaches, whereas contact time and initial concentration occupied ranks two to four depending on the method, whereas the relative importance of initial MG concentration, adsorbent dosage, temperature, and SDBS dosage varied according to the modeling approach. BR provided interpretable posterior estimates, with positive effects for pH and contact time and a negative effect for initial MG concentration, while variables with broader credible intervals, particularly temperature, adsorbent dosage, and SDBS dosage, were estimated with lower precision. RF demonstrated stronger internal predictive performance based on OOB evaluation; however, this performance was not maintained under more stringent validation schemes. Under group-aware leave-one-series-out validation, RF achieved R^2^ = −0.382, RMSE = 32.812 pp, and MAE = 22.124 pp, whereas BR yielded R^2^ = −124.886, RMSE = 313.213 pp, and MAE = 123.270 pp. On the independent held-out pH series, BR achieved R^2^ = 0.410, RMSE = 21.001 pp, and MAE = 17.426 pp, while RF achieved R^2^ = −0.911, RMSE = 37.793 pp, and MAE = 29.614 pp. These results demonstrate that stronger internal predictive performance does not necessarily translate into generalization across experimental series or operating regions not represented during model development. Overall, the findings support the complementary use of LMG, BR, and RF for interpreting the investigated adsorption dataset, while emphasizing that independent experimental validation is required before extrapolation to untested operating conditions. Characterization by SEM-EDS, FT-IR, XRD, TGA, and zeta potential analysis confirmed the hydroxyapatite-rich structure of SDBS-OBW and provided evidence for the presence of SDBS on the bone surface. The point of zero charge (PZC) sequence (raw OBW = 7.8, SDBS-OBW = 6.1, MG-loaded SDBS-OBW = 8.4) provides direct evidence for electrostatic attraction between the anionic sulfonate groups and the cationic MG^+^ species under the investigated conditions. For the specific system investigated, the results suggest that control of pH, contact time, and influent dye concentration should be prioritized over secondary variables such as temperature, SDBS dosage, and adsorbent dosage when optimizing removal efficiency within the studied ranges. The integrated LMG–BR–RF framework therefore offers a methodological approach for simultaneous variable-importance assessment, uncertainty quantification, and prediction in batch adsorption studies. Its extension to other systems, continuous-flow operation, or larger-scale applications would require additional experimental validation and is beyond the scope of the present work.

The conclusions of this study are further bounded by the following limitations:

(1) Batch operation only. All experiments were conducted in batch mode. The results do not directly translate to continuous-flow systems, packed-bed columns, or other operational configurations. Reactor sizing, hydraulic retention time, and scale-up cannot be inferred from the present data.
(2) Single model system. The integrated modeling framework was demonstrated on one system only (MG onto SDBS-OBW). Its transferability to other dyes, other adsorbents, or other water matrices was not tested and remains a hypothesis for future work.
(3) Limited dataset size. Fifty-two experimental conditions, arranged in six one-variable-at-a-time series whose runs are serially correlated (and therefore not statistically independent), were investigated, with each condition measured in triplicate (n = 3). The mean removal efficiency of the three replicate measurements was used as the response variable in the modeling dataset; thus, the analytical dataset contained 52 condition-level observations.
(4) One-variable-at-a-time design: importance rankings and cross-series predictions are confounded with series-specific background conditions (e.g., dosage, pH and temperature differ between series).
(5) No sustainability assessment. The present study does not include a life-cycle assessment, cost analysis, energy balance, or regeneration study. Consequently, the sustainability of SDBS-OBW as an adsorbent cannot be asserted on the basis of the present data. The use of a waste-derived precursor and a commercially available surfactant is consistent with circular-economy principles, but this does not constitute proof of sustainability.
(6) Partial mechanistic evidence. The PZC and pKa data provide direct evidence for electrostatic attraction. However, the proposed contributions of π–π stacking and hydrophobic interactions are inferred from indirect evidence and were not directly demonstrated by spectroscopic or calorimetric methods. The specific binding mode of SDBS on the hydroxyapatite surface was not determined.
(7) No economic analysis. The cost of SDBS modification, adsorbent preparation, and regeneration was not evaluated. Economic feasibility cannot be assessed from the present data.

## Figures and Tables

**Table 1:** Comparative importance ranking of the adsorption variables obtained from the LMG, Bayesian regression (normalised |posterior β|), and Random Forest (permutation importance, increase in MSE, normalised to 100%) models.

Variable	LMG (%)	LMG Rank	BR (%)	BR Rank	RF (%)	RF Rank
pH	31.48	1	29.12	1	35.04	1
Contact time	31.05	2	20.91	3	23.79	2
Initial concentration (C_0_)	25.91	3	23.19	2	15.23	4
Temperature	4.84	4	10.61	4	1.40	6
Adsorbent dosage	4.17	5	8.83	5	22.54	3
SDBS dosage	2.55	6	7.34	6	2.01	5

**Table 2:** Posterior coefficient estimates, standard deviations and 95% credible intervals obtained from Bayesian regression on the z-standardised scale, with convergence diagnostics (split $\hat{R}$ and bulk ESS; 4 chains × 25,000 iterations, warm-up 5000, thin 2; 40,000 retained draws). Priors: β_j_|σ^2^ ~ N(0, 10^2^); σ^2^ ~ Inv-Gamma(0.001, 0.001).

Parameter	Mean	Median	SD	CI 2.5%	CI 97.5%	$\mathrm{Split}\;\hat{R}$	Bulk ESS
Intercept	0.000	0.001	0.099	−0.195	0.197	1.000	8000
pH	0.650	0.650	0.145	0.365	0.935	1.000	3077
Contact time	0.467	0.466	0.104	0.263	0.672	1.000	8000
Initial concentration (C_0_)	−0.517	−0.517	0.124	−0.759	−0.272	1.000	4444
SDBS dosage	0.164	0.163	0.108	−0.051	0.378	1.000	40,000
Adsorbent dosage	0.197	0.197	0.149	−0.096	0.488	1.000	8000
Temperature	0.237	0.237	0.172	−0.103	0.576	1.000	40,000
sigma2	0.511	0.496	0.114	0.335	0.774	1.000	8000

**Table 3:** Comparative predictive performance of BR and RF models for malachite green removal efficiency prediction.

Model	Validation Scheme	R^2^	RMSE (pp)	MAE (pp)
Bayesian Regression	Group-aware CV (leave-one-series-out, 6 folds) ^a^	−124.886	313.213	123.270
Random Forest	Group-aware CV (leave-one-series-out, 6 folds) ^a^	−0.382	32.812	22.124
Bayesian Regression	Independent held-out test set (entire pH series; trained on the other five series) ^b^	0.410	21.001	17.426
Random Forest	Independent held-out test set (entire pH series; trained on the other five series) ^b^	−0.911	37.793	29.614
RF	OBB	0.822	11.79	7.85
BR	Leave-one-out CV	0.417	21.31	16.42

^a^ leave-one-series-out cross-validation: each of the six experimental series is held out once and predicted by the model trained on the other five series. ^b^ the pH series (10 runs, pH 2–11) was selected a priori as the independent test set; the five remaining single-series rotations are reported in Table S4.

## Data Availability

The minimal datasets supporting the TG and XRD analyses have been provided as Supplementary Materials. Additional raw data supporting the conclusions of this article are available from the corresponding author upon reasonable request.

## Supplementary Materials

The following supporting information can be downloaded at: https://www.mdpi.com/article/10.3390/molecules31193484/s1, Table S1. Complete specification of the Bayesian regression workflow and summary of the posterior-predictive checks. Table S2. The results of the EDS analysis for SDBS-OBW. Table S3. A summary of definite functional groups for raw OBW, SDBS-OBW, and MG-loaded SDBS-OBW. Table S4. Single-series independent test rotations: each OVAT experimental series is held out in turn and predicted by a model trained on the other five series. This is the strictest available test because the series are the natural independent blocks of the design. Figure S1. MCMC diagnostics of the Bayesian regression: trace plots of the four chains after warm-up (left column) and posterior distributions with posterior means and 95% credible intervals (right column) for the intercept, the six standardised predictors and the residual variance σ2. Visual stationarity and chain mixing correspond to split $\hat{R}$ ≤ 1.0001. Figure S2. Posterior-predictive checks of the Bayesian regression model: (left) kernel density of the observed removal efficiencies (black) overlaid with 120 of the 2000 posterior-predictive replicates (blue); (right) observed values against their 95% posterior-predictive intervals (coverage 96.2%). p_sd = 0.68, p_min = 0.32, p_max = 0.99. Figure S3. Stability of performance estimates across validation designs. Box plots of R2 and RMSE (pp) for BR (top row) and RF (bottom row): row-wise 10-fold CV (50 repeats), row-wise 75/25 hold-out (20 repeats), group-aware 3-fold CV (300 repeats), leave-one-series-out CV (pooled) and single-series hold-outs (6). Y-axis scales differ.
