# Supplementary material for: Integrated Machine Learning and Statistical Interpretation of Malachite Green Removal by SDBS-Modified Ostrich Bone Waste: A Comparative Random Forest, Bayesian Regression, and LMG Analysis
Source: Molecules. 2026 Sep 29;31(19):3484. doi: 10.3390/molecules31193484 (PMC13666174; doi:10.3390/molecules31193484)
Supplement: Supplementary file 1 [file molecules-31-03484-s001.zip › molecules-4553571-supplementary.pdf]

Supplementary Material- S1

Table S1. Complete specification of the Bayesian regression workflow and summary of the posterior-predictive checks.

Item	Specification
Likelihood	$y_i \sim N(x_i^T\beta, \sigma^2)$; y and predictors z-standardised within every training split
Prior, coefficients	$\beta_j \mid \sigma^2 \sim Normal(0, 10^2)$, j = intercept + 6 predictors (weakly informative)
Prior, residual variance	$\sigma^2 \sim$ Inverse-Gamma($a_0 = 0.001$, $b_0 = 0.001$)
Sampler	Gibbs sampling (exact conjugate conditional posteriors)
Chains	4 independent chains (different random seeds)
Iterations	25,000 per chain; warm-up 5,000 (discarded); thin = 2
Retained draws	4 × 10,000 = 40,000
Convergence	split $\hat{R} \leq 1.0001$ for all parameters; minimum bulk ESS ≈ 3,077
Posterior R^2	$1 - \sigma^2/Var(y\_std) = 0.489$; 95% CrI [0.226, 0.665]
PPC statistics	p_sd = 0.68; p_min = 0.32; p_max = 0.99 (2,000 replicated data sets)
PPI coverage	96.2% of observations inside their 95% posterior-predictive interval (2 of 52 outside)
Software	Python 3.13; scikit-learn 1.6.1 (RF); custom Gibbs sampler (BR); master seed 42 NumPy, SciPy, Matplotlib 3.10.9

Supplementary Material- S2

Detailed Characterization of SDBS-Modified Ostrich Bone Waste (SDBS-OBW)

S2.1 SEM–EDS characterization

Scanning electron microscopy coupled with energy-dispersive X-ray spectroscopy (SEM–EDS) was used to examine the surface morphology and elemental composition of SDBS-OBW. The EDS results reported in the characterization dataset showed carbon (18.17 wt.%), oxygen (15.38 wt.%), phosphorus (18.86 wt.%), and calcium (25.95 wt.%) as the major detected elements. Nitrogen (8.97 wt.%), sodium (2.51 wt.%), magnesium (1.84 wt.%), and sulfur (2.83 wt.%) were also detected, while Al (1.19 wt.%), Cr (1.33 wt.%), Mn (1.34 wt.%), and Fe (1.62 wt.%) occurred at lower concentrations. The Ca- and P-rich composition is consistent with the hydroxyapatite-rich inorganic framework expected for bone-derived materials. The presence of sulfur provides supporting evidence for the SDBS-derived sulfonate component, while sodium is compatible with the sodium counterion associated with SDBS. The carbon and nitrogen signals are consistent with residual organic/proteinaceous constituents. The SEM image shows an uneven, folded and heterogeneous surface with an apparent porous structure, indicating potentially accessible adsorption sites. These observations are consistent with previous studies of surfactant-modified ostrich bone waste, in which chemical modification increased the accessibility of surface sites and produced bio-adsorbents suitable for aqueous contaminant removal.

Table S2. The results of the EDS analysis for SDBS-OBW

Elt.	Line	Intensity (c/s)	Atomic %	Conc	Units
C	Ka	1.06	31.73	18.17	wt.%
N	Ka	0.18	13.44	8.97	wt.%

O	Ka	0.83	20.17	15.38	wt.%	
Na	Ka	0.85	2.29	2.51	wt.%	
Mg	Ka	0.88	1.59	1.84	wt.%	
Al	Ka	0.66	0.92	1.19	wt.%	
P	Ka	12.00	12.77	18.86	wt.%	
S	Ka	1.59	1.85	2.83	wt.%	
Ca	Ka	13.91	13.58	25.95	wt.%	
Cr	Ka	0.46	0.54	1.33	wt.%	
Mn	Ka	0.41	0.51	1.34	wt.%	
Fe	Ka	0.44	0.61	1.62	wt.%	
			100.00	100.00	wt.%	Total

Table S3. A summary of definite functional groups for raw OBW, SDBS-OBW, and MG-loaded SDBS-OBW

Wavenumber (cm^{-1})	Functional Group	Vibrational Mode	Raw OBW	SDBS-OBW	MG-Loaded SDBS-OBW
3300–3500	O–H	ν(O–H) stretching	Present	Present and shifted	Present (broader)
2920–2850	C–H	ν_{as}(C–H), ν_s(C–H)	Strong	Strongly reduced	Weak
1650	Amide I (collagen), Aromatic C=C (MG)	ν(C=O) + ν(C=C) + ν(C=N)	Present	Decreased	Strong / Enhanced
1540	Amide II (collagen) + Aromatic C=C (MG)	δ(N–H) + ν(C–N) + ν(C=C)	Present	Decreased	Prominent
1450	CO_3^{2-} + Aromatic C=C (MG)	$\nu_3(CO_3^{2-})$+ ν(C=C)	Present	Present	Strong, broad t
1415	CO_3^{2-}+ Aromatic C=C (MG	$\nu_3(CO_3^{2-})$	Present	Present	strong
1400–1600	Aromatic C=C, C=N (MG) + N–H bending (MG)	ν(C=C), ν(C=N) δ(N–H)	Weak	Weak	strong
1000–1100	PO_4^{3} (hydroxyapatite) + Sulfonate S=O	$\nu_3(PO_4^{3-})$ asymmetric stretching	Strong	Present	Broad,
960	PO_4^{3-}	$\nu_1(PO_4^{3-})$ symmetric stretching	Present	Present	Present
603	PO_4^{3-}	$\nu_4(PO_4^{3-})$ bending	Present	Present	strong
560	PO_4^{3-}	$\nu_4(PO_4^{3-})$ bending	Present	Present	strong

Supplementary Material- S3

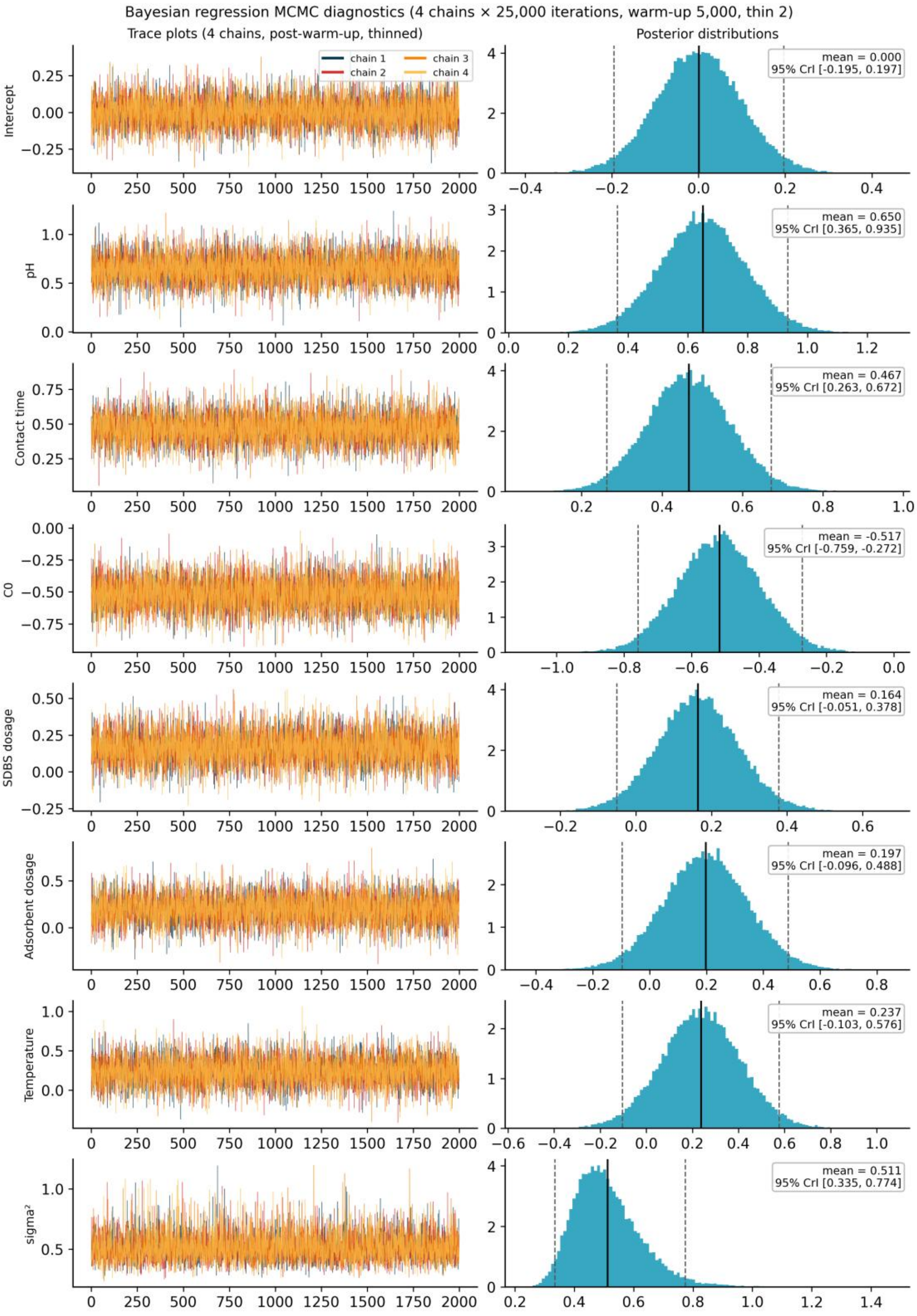

Figure S1. MCMC diagnostics of the Bayesian regression: trace plots of the four chains after warm-up (left column) and posterior distributions with posterior means and 95% credible intervals (right column) for the intercept, the six

standardised predictors and the residual variance σ². Visual stationarity and chain mixing correspond to split $\hat{R} \leq 1.0001$.

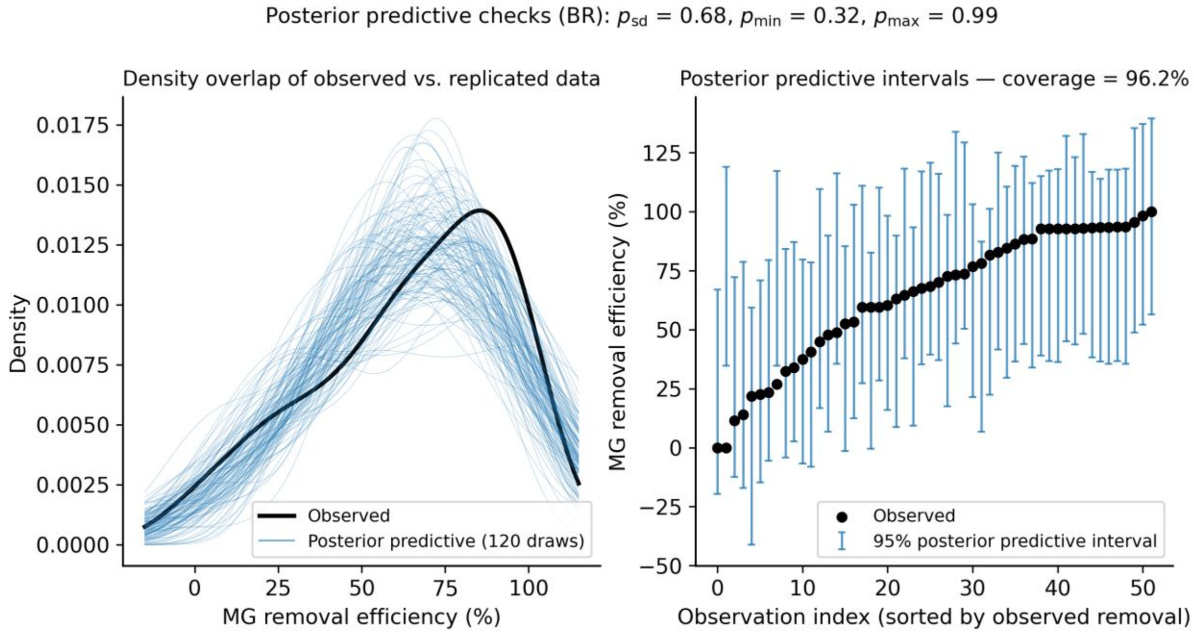

Figure S2. Posterior-predictive checks of the Bayesian regression model: (left) kernel density of the observed removal efficiencies (black) overlaid with 120 of the 2,000 posterior-predictive replicates (blue); (right) observed values against their 95% posterior-predictive intervals (coverage 96.2%). p_sd = 0.68, p_min = 0.32, p_max = 0.99.

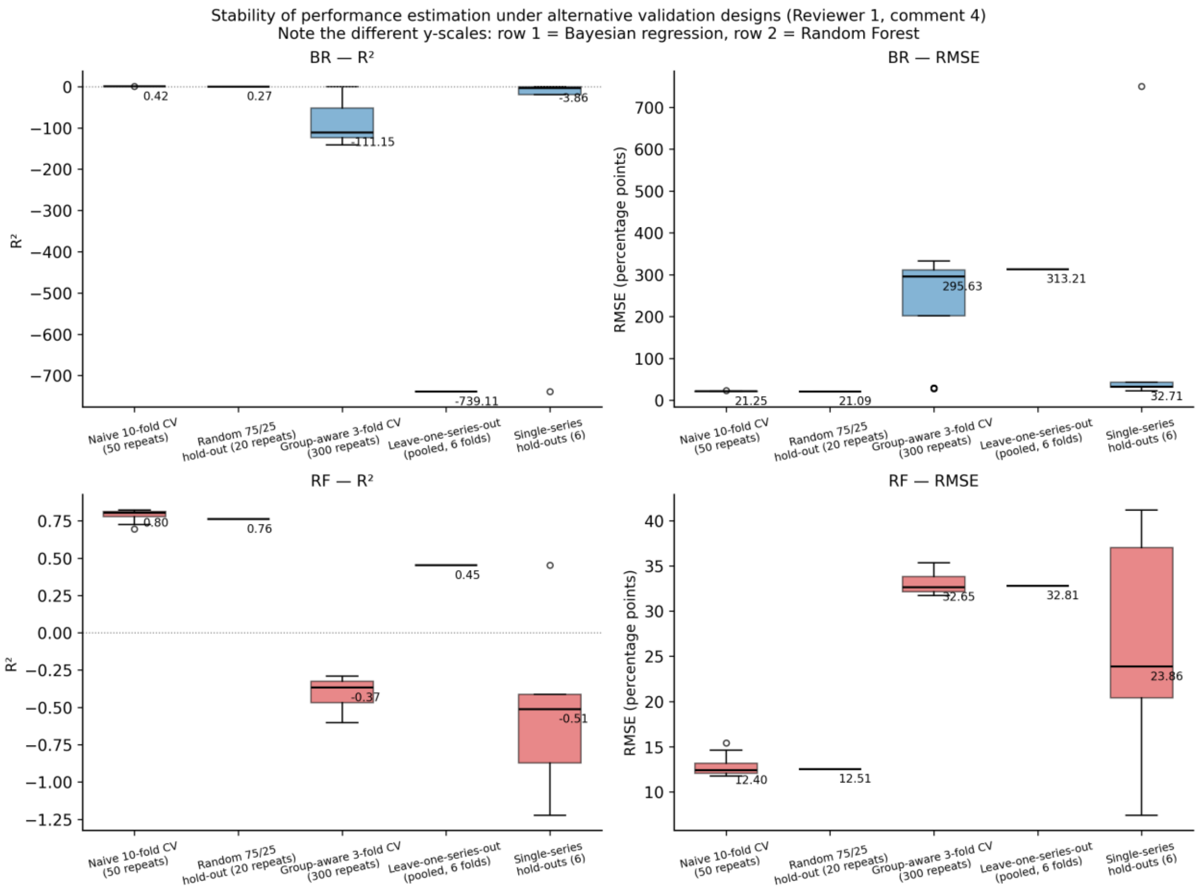

Figure S3. Stability of performance estimates across validation designs. Box plots of R^2 and RMSE (pp) for BR (top row) and RF (bottom row): row-wise 10-fold CV (50 repeats), row-wise 75/25 hold-out (20 repeats), group-aware 3-fold CV (300 repeats), leave-one-series-out CV (pooled) and single-series hold-outs (6). Y-axis scales differ.

Table S4. Single-series independent test rotations: each OVAT experimental series is held out in turn and predicted by a model trained on the other five series. This is the strictest available test because the series are the natural independent blocks of the design.

Held-out series (independent test)	Model	R^2	RMSE (pp)	MAE (pp)
pH (10 runs)	BR	0.410	21.001	17.426
time (11 runs)	BR	-0.088	31.400	24.519
C0 (9 runs)	BR	-739.107	749.775	595.830
SDBS (7 runs)	BR	-3.860	42.784	38.129
Dose (11 runs)	BR	-0.102	32.715	22.828
T (4 runs)	BR	-19.290	22.419	21.396
pH (10 runs)	RF	-0.911	37.793	29.614
time (11 runs)	RF	-0.873	41.195	28.705
C0 (9 runs)	RF	0.452	20.394	15.084
SDBS (7 runs)	RF	-0.511	23.858	20.353
Dose (11 runs)	RF	-0.413	37.034	21.401
T (4 runs)	RF	-1.222	7.419	6.222
